# Causes of Performance Degradation in Non-invasive Electromyographic Pattern Recognition in Upper Limb Prostheses

**DOI:** 10.3389/fnbot.2018.00058

**Published:** 2018-09-21

**Authors:** Iris Kyranou, Sethu Vijayakumar, Mustafa Suphi Erden

**Affiliations:** ^1^Edinburgh Centre of Robotics, Edinburgh, United Kingdom; ^2^School of Informatics, Institute of Perception, Action and Behaviour, University of Edinburgh, Edinburgh, United Kingdom; ^3^School of Engineering and Physical Sciences, Heriot-Watt University, Edinburgh, United Kingdom

**Keywords:** upper limb prostheses applications, electromyography, EMG drifts, EMG concept drift, EMG variability with time, EMG variability between users

## Abstract

Surface Electromyography (EMG)-based pattern recognition methods have been investigated over the past years as a means of controlling upper limb prostheses. Despite the very good reported performance of myoelectric controlled prosthetic hands in lab conditions, real-time performance in everyday life conditions is not as robust and reliable, explaining the limited clinical use of pattern recognition control. The main reason behind the instability of myoelectric pattern recognition control is that EMG signals are non-stationary in real-life environments and present a lot of variability over time and across subjects, hence affecting the system's performance. This can be the result of one or many combined changes, such as muscle fatigue, electrode displacement, difference in arm posture, user adaptation on the device over time and inter-subject singularity. In this paper an extensive literature review is performed to present the causes of the drift of EMG signals, ways of detecting them and possible techniques to counteract for their effects in the application of upper limb prostheses. The suggested techniques are organized in a table that can be used to recognize possible problems in the clinical application of EMG-based pattern recognition methods for upper limb prosthesis applications and state-of-the-art methods to deal with such problems.

## 1. Introduction

Over the last years the design of prosthetic devices has evolved incorporating electrically actuated components in conjunction with the classic mechanical design. Modern prosthetic hands, like i-Limb by Touch Bionics^1^, BeBionic^2^, and Vincent hand^3^ consist of five, individually actuated digits and use myoelectric techniques for their control. Novel control methods become necessary that allow to take full advantage of the functionalities of the new devices.

In this direction, myoelectric control techniques were investigated and employed for the control of the prosthetic devices. A myoelectric-controlled prosthesis records the electrical signals generated by the remaining muscles of the patient and utilizes them to control the prosthetic limb. Commercial prostheses' companies utilize myoelectric control by training the patients to trigger specific muscle signals that are used to access a grip. This technique is very robust, but not intuitive and limited by the patient's ability to remember and perform the trigger motions, potentially leading to the abandonment of the device (Biddiss and Chau, 2007).

In an attempt to reduce the mental load of the patient and provide a more intuitive control of upper limb prosthetic devices, pattern recognition methods have been extensively investigated over the last few decades. The input to the classifier in this case would be the electromyographic signals and the output would be a class corresponding to the intended grasp. Academic research has focused on training different classifiers, including probabilistic model algorithms, such as linear discriminant analysis (LDA) (Chen et al., 2011; Zhang et al., 2012; Phinyomark et al., 2013a; Young et al., 2013; Liu et al., 2016a) and hidden Markov models (HMM) (Chan and Englehart, 2005); support vector machines (SVM) (Bitzer and van der Smagt, 2006; Lucas et al., 2008; Castellini and van der Smagt, 2009; Alkan and Günay, 2012), artificial neural networks (ANN) (Ahsan et al., 2011) and more recently deep learning convolutional networks (CNN) (Atzori et al., 2016; Wei et al., 2017; Zhai et al., 2017; Côté-Allard et al., 2018) to recognize the intended pre-grasp. The investigated classifiers were able to distinguish between four to 53 different grasp classes, achieving high performance, which often exceeds 90% accuracy (Putnam and Knapp, 1993; Christodoulou and Pattichis, 1999; Kim et al., 2004; Lucas et al., 2008; Scheme et al., 2011; Zhang et al., 2012; Ortiz-Catalan et al., 2013).

In order to improve the performance of classifiers even further, a big amount of research has focused on identifying the most suitable feature sets that will provide higher accuracy and more robust performance (Phinyomark et al., 2012a; Shin et al., 2014; Adewuyi et al., 2016). Alternatively, researchers have attempted to increase the classifier's performance by incorporating extra sensors, like accelerometers, magnetometers, gyroscopes and cameras (Fougner et al., 2011a; Gijsberts et al., 2014a; Kyranou et al., 2016; Krasoulis et al., 2017). Other techniques to improve the classifier's performance is adding a dimensionality reduction step in the preprocessing of the data, like independent component analysis (ICA), principle component analysis (PCA) or nonnegative matrix factorization (NMF) (Naik and Nguyen, 2015; Naik et al., 2016; Zhang et al., 2017).

However, although high classification accuracies have been reported in offline analysis of experiments performed under controlled laboratory conditions, these myoelectic pattern recognition methods are not widely used in clinical applications. Only recently was a real-time pattern recognition control system introduced commercially^4^, but it used EMG data to classify only between open and close gestures and wrist rotation motion. Many recent studies pointed out that there is no direct correlation between offline analysis performance improvement and online (real-time) performance (Jiang et al., 2014; Ortiz-Catalan et al., 2015; Vujaklija et al., 2017), emphasizing the need for online evaluation of such systems.

Moreover, when it comes to long-term myoelectric pattern recognition systems a big challenge is that the EMG signal is non-stationary in nature and its statistical properties change over time. This results in control systems that are unstable or difficult to use after a period of time (Kwatny et al., 1970; Park et al., 2016). The most common causes of this variability of the EMG signal include physiological reasons, such as muscle fatigue, muscle atrophy or hypertrophy, electrode conductivity (perspiration, humidity); user variations due to adaptation or learning; and physical reasons, such as electrode shift, soft tissue fluid fluctuations, contraction intensity changes between trials, additional weight and arm posture change (Sensinger et al., 2009).

In this paper we perform a literature review that describes the most common reasons causing the EMG signal variability, the different methods that are utilized to detect them, and the solutions proposed to mitigate their effect on the EMG signal. The reviewed models are discussed along with applications that utilize them to improve the performance of upper-limb prostheses. Accordingly, in Table 1, one can follow which literature addresses which causes of EMG signal drift, whether they provide a method for detection of the drift, a model to explain the drift and what kind of approaches they follow to mitigate the causes of the signal drift.

**Table 1:** Causes of EMG drifts; how to detect and model them and approaches to mitigate them.

**Cause**	**Research**	**Detection**	**Model**	**Applications**		
				**Data abundance**	**Feature set**		
Fatigue	Stulen and Luca, 1981	+	+		+		
	De Luca and Van Dyk, 1975; De Luca, 1983	+	+		+		
	Merletti et al., 1984, 1990	+	+		+		
	Park and Meek, 1993		+		+		
	Enoka and Duchateau, 2007		+		+		
	Luttmann et al., 2000	+		+			
	Castellini et al., 2009	+		+			
	Artemiadis and Kyriakopoulos, 2008, 2010	+			+		
	Mainardi et al., 2008	+			+		
	Song et al., 2006				+		
	Phinyomark et al., 2012c						
	Cifrek et al., 2009				+		
	Cao et al., 2007				+		
	Al-Mulla et al., 2009; Al-Mulla and Sepulveda, 2010	+					
	Tkach et al., 2010	+					
	Ravier et al., 2005	+					
				**Data abundance**	**Feature set**	**Electrode configuration**	
Electrode displacement	Hudgins et al., 1993	+					
	Hargrove et al., 2006, 2008	+	+	+	+		
	Tkach et al., 2010		+		+		
	Young et al., 2012	+	+		+	+	
	Boschmann and Platzner, 2012, 2014	+	+	+			
	Muceli et al., 2014			+		+	
	Fang and Liu, 2014					+	
	Stango et al., 2015			+	+	+	
	Pan et al., 2015			+	+	+	
				**Data abundance**	**Feature set**	**Additional sensors**	
Arm posture	Scheme et al., 2010	+		+		+	
	Chen et al., 2011	+		+			
	Fougner et al., 2011b	+		+		+	
	Liu et al., 2012, 2014	+		+	+		
	Khushaba et al., 2012, 2016	+		+	+	+	
Arm posture (*Continued*)	Geng et al., 2012	+		+		+	
	Jiang et al., 2013	+		+			
	Boschmann and Platzner, 2014	+					
	Yang et al., 2015	+					
	Betthauser et al., 2016	+					
				**Simultaneous learning**	**Transferable skills**		
Learning	Nishikawa et al., 2000			+			
	Yokoi et al., 2004	+		+			
	Mosier et al., 2005				+		
	Kato et al., 2006a,b	+		+			
	Zhang et al., 2008	+					
	Radhakrishnan et al., 2008				+		
	He et al., 2013	+					
	Pistohl et al., 2013				+		
	Antuvan et al., 2014				+		
	Powell et al., 2014	+					
	He et al., 2015	+	+				
	Ison et al., 2014, 2016; Ison and Artemiadis, 2015	+	+		+		
			**Update model**	**Data abundance**	**Feature se**t	**Adaptation**	**Prosthesis guided learning**
Concept drift	Fukuda et al., 1997, 2003	+	+			+	
	Bitzer and van der Smagt, 2006	+		+			
	Sensinger et al., 2009	+	+				
	Kaufmann et al., 2010	+		+			
	Artemiadis and Kyriakopoulos, 2011	+		+			
	Jain et al., 2012	+	+	+		+	
	Amsuss et al., 2013	+					
	Liu, 2015	+	+	+		+	
	Liu et al., 2016b	+	+				
	Tkach et al., 2010						
	Zhang et al., 2008		+		+		
	Phinyomark et al., 2012c, 2013b				+		
	Gijsberts et al., 2014b		+			+	
	Vidovic et al., 2016					+	
	Lock et al., 2011						+
	Simon et al., 2012						+
Concept drift (*Continued*)	Chen et al., 2013		+			+	
	Du et al., 2017		+			+	
	Zhai et al., 2017		+			+	
				**Data abundance**	**Feature set**	**Adaptation**	
Inter-subject variability	Orabona et al., 2009			+		+	
	Chattopadhyay et al., 2011				+		
	Matsubara et al., 2011; Matsubara and Morimoto, 2013			+	+	+	
	Gibson et al., 2013			+			
	Ison and Artemiadis, 2013			+	+		
	Tommasi et al., 2013			+			
	Khushaba, 2014			+	+	+	
	Phinyomark et al., 2013b			+	+	+	
	Guo et al., 2015			+	+		
	Stival et al., 2016			+			

Note here that a lot of fluctuations in EMG signals are the result of sensor noise that can be reduced or eliminated with improved hardware design (Mainardi et al., 2008; Huang et al., 2010; Hahne et al., 2016; Yokus and Jur, 2016). We do not investigate such issues that pertain to the design development and quality of equipment; rather we focus on disturbances that add noise over time because of a change in the framework or physiology of the user and physical impacts external to the device.

Finally, in this paper we focus on non-invasive applications that utilize surface EMG readings. Pattern-recognition techniques, like targeted muscle reinnervation (TMR) (Zhou et al., 2007; Kuiken et al., 2009), that couple a surgical reinnervation procedure with surface EMG are still prone to some of the disturbances that are analyzed in the following work, like muscle fatigue or electrode displacement. Moreover, due to the nature of the procedure, they introduce interferences that are not present in EMG signals recorded from the remaining arm of a transradial amputee, like electrocardiography (ECG) interference (Hargrove et al., 2009). Such interferences that are specific to TMR procedure are not to be investigated in the following work.

## 2. Causes of EMG variability with time

In our literature review we have identified five major causes of surface EMG signal changes that affect the pattern-recognition performance, namely,

1. muscle fatigue,
2. electrode shifts,
3. arm posture,
4. learning/adaptation of user, and
5. inter-subject variability.

Following, we review these by focusing on the cause of each problem, the models that are proposed to formulate the cause and effect, and the computational techniques used to mitigate their effect.

### 2.1. Muscle fatigue

The term muscle fatigue is used to describe a temporary decrease in one's physical capacity of performing motions. The development of muscle fatigue is typically quantified as a decline in the maximal force or power capacity of the muscle, thus resulting in different signal recordings from the EMG electrodes over time (Enoka and Duchateau, 2007). In most cases of myoelectric pattern recognition applications in experimental environments researchers are trying to avoid the presence of fatigue by limiting the duration of a trial and allowing enough resting time between trials. However this is not feasible in a real-time scenario of constant usage of a prosthetic device.

In an attempt to model the impact that fatigue has on the EMG signals, different EMG signal features and characteristics were investigated, like signal amplitude and the power spectrum density (PSD). Early research reported an increase in myoelectric signal amplitude (Cobb and Forbes, 1923; Stulen and Luca, 1981; Merletti et al., 1990; Park and Meek, 1993) when subjects were holding for several seconds an isometric muscle contraction. However, many researchers observed that the amplitude alone is not a sufficient metric to determine the presence of fatigue, since an increase in ME amplitude is also observed in other cases, like when greater force is applied in the manipulation of an object (Ravier et al., 2005). Additionally to the aforementioned amplitude increase, a shift toward the lower frequencies of the ME signal power spectrum is observed in fatigued muscle recordings (Cobb and Forbes, 1923; Lindstrom et al., 1970; Stulen and Luca, 1981; De Luca, 1983; Merletti et al., 1990; Park and Meek, 1993). De Luca (1983) reported a possible decrease of the mean- or median-frequencies by more than 50% in value from the beginning to the end of a sustained isometric constant-force contraction. However, the amount of decrease appears to be dependent on the muscle under investigation. Park and Meek (1993) found a correlation between EMG magnitude and frequency shifts; more specifically the EMG magnitude does not increase until the median frequency of the ME signal decreases to a certain level.

The amplitude increase and the frequency shift of EMG signals can be fairly well explained by the muscle conduction velocity changes alone (Basmajian and De Luca, 1985; Park and Meek, 1993). Lindstrom et al. (1970) have developed a general mathematical model of the EMG power spectrum density (PSD) and have shown that both the amplitude increase and the spectral shift toward lower frequencies can be explained by the conduction velocity changes during a sustained contraction. Furthermore, the characteristic frequencies of the EMG PSD, such as mean and median frequencies, are linearly proportional to the conduction velocity (Stulen and Luca, 1981; De Luca, 1983; Arendt-Nielsen and Mills, 1985; Merletti et al., 1990; Park and Meek, 1993), a linear relationship that was proven by Sadoyama et al. (1983).

#### 2.1.1. Detection

Detection of fatigue relies on identifying the features that measure the aforementioned EMG signal frequency and amplitude shifts. Kwatny et al. (1970) was the first to introduce the mean frequency (MNF) of the ME spectrum as a suitable metric to describe such spectrum shifts to detect fatigue. Mean frequency along with median frequency (MDF) metric were the most popular metrics associated with the decrease in frequency in the fatigued state (Stulen and Luca, 1981; De Luca, 1983; Merletti et al., 1984; Park and Meek, 1993; Song et al., 2006; Mainardi et al., 2008; Phinyomark et al., 2012c; Thongpanja et al., 2013). Song et al. (2006) suggested a rule that indicates the presence of fatigue when the MNF and MDF values are less than specific thresholds. On the other hand, De Luca (1983) defined the failure point in time by monitoring force; lack of maintaining the desirable level of force output indicates the switch to a fatigued state.

**Figure 1:**
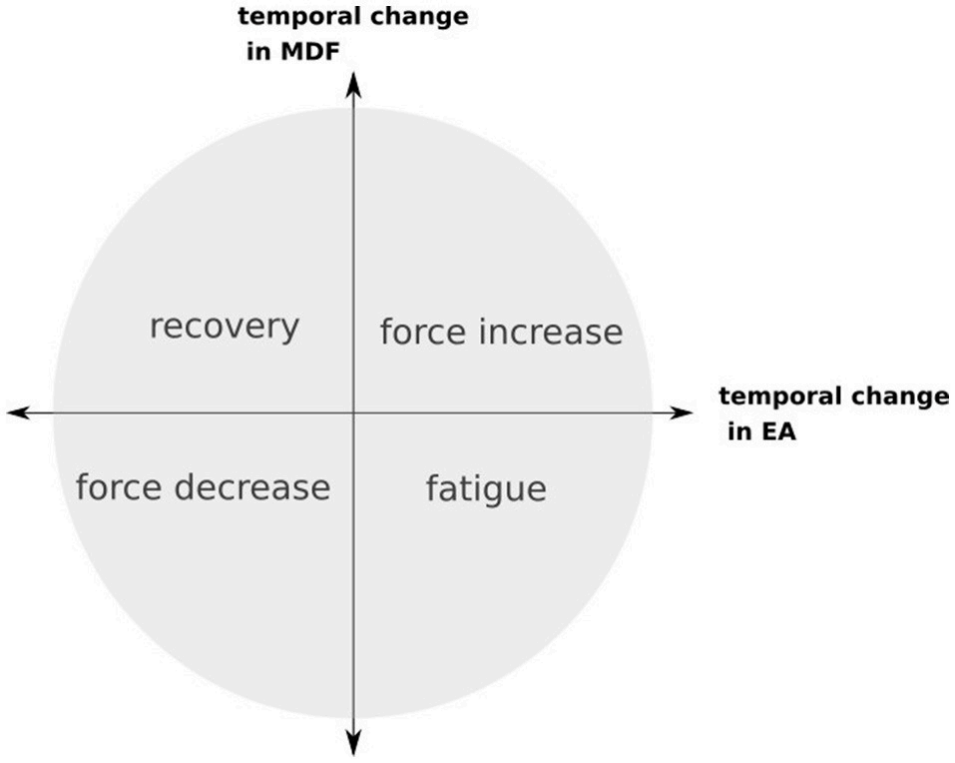
Graph representing algorithm in Luttmann et al. (2000). Increase of with shift of median frequency (MDF) to the higher frequencies corresponds to force increase whereas increase in amplitude and shift to the lower frequencies indicates muscle fatigue. Similarly a decrease of the with simultaneous shift to the lower frequencies of the median frequency indicates force decrease whereas shift to the higher frequencies recovery from fatigue.

Luttmann et al. (2000) combined the information about the myoelectric signal's behavior in time and frequency domain and described a simple four-case algorithm that looks at changes both at and frequency shift, in order to decide whether these changes are a result of force increase or decrease, muscle fatigue or recovery from fatigue. When the increase in myoelectric amplitude is also followed by a decrease in the signal's frequency then the muscle is in a fatigued state (see Figure 1).

Various different features have been investigated to assess muscle fatigue such as wavelet transform (Kumar et al., 2003; Cao et al., 2007; Camata et al., 2010; Bartuzi and Roman-Liu, 2014), the number of zero crossings (Hägg, 1981; Masuda et al., 1982) and autoregressive coefficients (Inbar et al., 1986; Paiss and Inbar, 1987; Al-Mulla et al., 2009). Bonato et al. (2001) used the time-frequency parameters of instantaneous mean and median frequencies (IMNF and IMDF). Al-Mulla et al. (Al-Mulla and Sepulveda, 2010) have created a new feature, called 1D spectro_std, which is defined as the standard deviation of a unified signal consisting of the instantaneous median frequency and the total band power, in order to detect between three different levels of fatigue, namely Non-Fatigue, Transition-to-Fatigue and Fatigued. Their classifier achieves average offline accuracy increase of 20.58% in comparison to just the instantaneous median frequency, the total band power, a spectral index and Wavelet decomposition. Tkach et al. (2010) have compared different features' performance in the classification of four types of isometric contractions. They concluded that the most stable feature set in the case of fatigue is consisted of the combination of Waveform Length, Slope Sign Changes (slopeSign), Autoregression (AR) and Cepstrum coefficients (Ceps) features, which resulted in 85.6 ± 4.8% accuracy across subjects when training data were recorded from rested muscles and test data from fatigued muscles. More extensive references to the different features used in literature to access muscle fatigue are presented by Cifrek et al. (2009).

Another approach is to train a classifier to recognize the presence and level of fatigue. Artemiadis and Kyriakopoulos (2008) proposed a probabilistic framework that assigns to each of the recorded muscles a class related to the fatigue level, in order to implement a control interface to manipulate a robotic arm in real-time. They trained a different model for discrete levels of fatigue and used this time-varying switching model to compensate for the EMG changes. In a later study the authors introduced the median frequency as an additional feature, in order to reinforce the detection of muscle fatigue (Artemiadis and Kyriakopoulos, 2010). In a similar manner Subasi and Kiymik (2010) trained a classifier that recognizes the presence of fatigue in the signal utilizing time-frequency features.

It is important here to note that along research the majority of features that are proposed for fatigue recognition are on the frequency domain, since these are capable of capturing the underlying biochemical phenomenon that manifests in the observed shift of the EMG spectrum toward lower frequencies.

#### 2.1.2. Approaches for mitigation of drift due to fatigue

In order to simulate the results of muscle fatigue, the subjects are instructed to perform a task repetitively or to hold an isotonic motion or grasp for a specific amount of time. The time that is perceived enough for the presence of fatigue varies and depends on the type of exercise and the subject's stamina. Most commonly it ranges between 30 s (Navaneethakrishna and Ramakrishnan, 2014) and up to 4 min (MacIsaac et al., 2001; Artemiadis and Kyriakopoulos, 2008; Castellini and van der Smagt, 2009). This allows enough time to be able to record data from non-fatigued and fatigued stages.

One of the first attempts to compensate the muscle fatigue effects was by Park and Meek (1993) who tried to counteract to the two effects of fatigue on the EMG signal; increase in the signal amplitude and shift toward the lower frequencies. They proposed a preprocessing method that scales down the EMG amplitude and utilizes a EMG power spectrum density (PSD) model, in order to decompress the fatigued EMG PSD to the unfatigued EMG PSD.

In a later study, Song et al. (2006) observed that feature variations are consistent for a duration time of muscle contractions and utilized this information to create a look-up table technique that allows to estimate the level of fatigue and use this information to adjust the min-max values of hyperboxes in a Fuzzy Min-Max Neural Network.

Knowledge on how the properties of the EMG signal change over time due to presence of fatigue was incorporated in the development of devices as well. Mainardi et al. (2008) designed a new EMG electrode suitable both for prosthetic control and frequency analysis, which allowed real-time modification of the electrode's gain, to compensate in case of muscular fatigue.

As mentioned previously, in order to mitigate for the effects of fatigue research focuses on collecting data from multiple levels of fatigue and utilizes the abundance of information. This requires recording more data than a simple classification case and creates different computational requirements to the system.

### 2.2. Electrode shift

Another important problem associated with everyday use of prosthetic devices, is electrode shifts. Donning/doffing or repositioning of the prosthetic socket may result in electrodes' displacement from their original position. This can happen over time during a single day or may refer to positioning the electrodes in slightly different positions from day to day, hence resulting in different recordings from the electrodes.

In order to simulate the effect of electrode shift in the experiments performed, multiple electrodes are placed in adjacent areas of the original locations of the electrodes (Hargrove et al., 2008; Boschmann and Platzner, 2012). The most common experimental procedure consists of training the classifiers on features extracted from the original signals and testing the classifier on features from the shifted signals. The maximum electrode displacement distance that is likely to occur in the normal everyday use of the prosthetic hand is noted in different studies as 1 cm (Hargrove et al., 2008; Boschmann and Platzner, 2012, 2014; Muceli et al., 2014; Pan et al., 2015; Stango et al., 2015).

#### 2.2.1. Detection

The detection of electrodes shifts from their original positions does not follow a physiological pattern that can be described by ME signal characteristics as in the case of muscle fatigue. Most studies rely on indirectly detecting the electrode shift by monitoring either the classification accuracy or the classification error (Hargrove et al., 2008; Tkach et al., 2010; Boschmann and Platzner, 2012, 2014; Young et al., 2012; Pan et al., 2015; Stango et al., 2015). A decrease in the former or increase in the latter is associated with some disturbance in the EMG signals, that is not necessarily uniquely associated with electrode shift and could be the result of other disturbances (see following sections).

Early research provided no evidence of significant effect of small electrode shift in the EMG recordings. Hudgins et al. (1993) found that shifts of up to 2 cm had relatively little effect on classification accuracy of a 5-class myoelectric control problem. In the experiment they conducted only two electrodes were used, one over the biceps brachii and one over triceps brachii muscles, which are antagonistic and with a big inter-electrode distance between them. This big distance could explain the higher distinctive property between signals acquired from these muscles. Their conclusion agrees with the observations of Young et al. (2012) that an increase in inter-electrode distance from 2 to 4 cm reduced the classification error and resulted in higher controllability [measured in terms of a virtual prosthesis control test, the Target Achievement Control (TAC) test (Simon et al., 2011)]. In this study four electrode sites were placed equidistantly in the subjects' forearms which, as with the Hudgins et al. (1993) study, resulted in high inter-electrode distance and more distinctive muscles targeted by the sensors.

However, the majority of studies have shown a correlation between electrode shifts and a decrease in classification accuracy, especially when a higher number of electrodes are used and more postures are classified. Hargrove et al. (2006) observed a reduction of approximately 30% in the classifier's performance with electrode shifts when they used five electrodes equally spaced around the forearm, and a drop in the classification accuracy of 6 and 9%, for the case of time-domain autoregressive (TDAR) features and time-domain (TD) features respectively in a later study (Hargrove et al., 2008). Similar conclusions on the drop in performance of EMG signal classification under the presence of electrode displacements are presented in various papers (Boschmann and Platzner, 2012; Young et al., 2012; Stango et al., 2015).

#### 2.2.2. Approaches of mitigation of drift due to misplacements

The basic approach in dealing with electrode displacement in literature is **data abundance**. Hargrove et al. (2008) used extra sensors that were placed on the hypothetical shifted positions and proved that when the classifier is trained over all displacement locations the classification error reduces in comparison to only training in the nominal positions. However, this strategy needs long-time training and can be frustrating for the user, thus potentially leading to frequent device abandonment.

Boschmann and Platzner (2012) achieved information abundance by incorporating a large set of electrodes; in this study 96 electrodes. Whenever a decline in classification accuracy was observed they eliminated the sensors that were evaluated as the ones being most responsible for the appearance of disturbances and retrained with the new subset of sensors. In their study they also investigated the maximum amount of sensors that are needed to compensate for a 1*cm* electrode shift and starting with 96 sensors they concluded that 32 sensors are sufficient to compensate the electrode displacement effect.

The sensor configuration that was used in this study, that consists of a large number of small sized electrodes (typically more than 16 electrodes) is called a high-density electromyography (HD-EMG) array and have been increasingly used in recent research (Daley et al., 2012; Hahne et al., 2012). The benefit of using HD-EMG arrays is that they cover a large area of skin with higher resolution than normal sensors, but, on the other hand, they require precise and efficient electronics design, both in matter of size and battery capacity.

Stango et al. (2015), similar to Boschmann and Platzner (2012), exploited the abundancy of information gathered from the the HD-EMG electrode arrays to perform an electrode selection technique and reduce the amount of sensors used in the testing phase of the classifier. This technique can be useful when the sensors that are most responsible for disturbances due to noise or displacement are chosen to be eliminated and the system is retrained with the rest of the sensors. While both studies succeeded in sustaining a high classification accuracy over time, their approach demands a retraining of the classifier each time a new subset of sensors is selected as optimal. In a real life case this would introduce a disruption in the use of the prosthesis for as long as the training lasts, which can result in confusion and frustration of the user.

López et al. (2009) investigated the performance of a single degree-of-freedom control comparing two different data fusion techniques. They suggest that utilizing more than a single recording from a specific set of muscles, even when the recordings are from slightly different positions that are then fused together, improves the robustness of the myoelectric control system.

All these methods require recording training data from multiple electrode displacement positions which is impractical in real life applications and increases the classifiers' computational load Muceli et al. (2014) suggested to focus on extracting signals that are robust to electrode displacement and electrode numbers. They used non-negative matrix factorization (NMF) technique as a semi-supervised way to extract signals and used them to control a prosthetic hand in real-time. They performed online and offline experiments on the electrode configuration and they didn't find significant difference between utilizing 6, 8 or 16 channels. Moreover their system does not need retraining, but only a calibration in the beginning of the experiment, which allows a more natural control of the device.

Different **electrodes' configurations** have been investigated in research, not only in terms of number of electrodes, but also in terms of variations in the electrode size and orientation. Young et al. (2012) demonstrated that electrodes with larger size reduced the sensitivity of shift, but they performed worse in comparison with smaller electrodes when no displacement was present, thus they did not find the larger electrodes more beneficial in practical applications. In the same study they suggested that electrodes oriented in the longitudinal direction with the muscle fibers performed better than the ones oriented in transversal direction, since the shift was mostly over the same muscle's fibers, so information about the muscle excitation was preserved. This observation is also discussed in the work by Stango et al. (2015) who used HD EMG signals around the subjects' forearms and detected smaller loss of classification accuracy in longitudinal shifts rather than shifts in transversal direction.

Experiments were also performed to deduce the **feature sets** that provide more robust classifiers in the presence of electrode shifts. Tkach et al. (2010) performed a comparative study between eleven commonly used time domain (TD) features and determined the set consisting of variance (var), ν-Order, log detector (logDetect), and EMG histogram (emgHist) features to be the most robust in the presence of electrode shifts. Young et al. (2012) showed that time-domain autoregressive (TDAR) features achieved the best real-time classification performance and was least affected by electrode displacements, in comparison with TD feature set. Similar results are seen in Hargrove et al. (2008), where the classification accuracy drops from 93 to 87% for the TDAR feature set and from 90 to 81% for the TD feature set. Stango et al. (2015) used an experimental measure of the degree of spatial correlation called variogram in their classification experiments and showed that it reduced sensitivity to electrode shifts compared with TD, RMS and TDAR features. Pan et al. (2015) found that multiclass common spatial patterns (CSP) performed better than the TD, TDAR and variogram features providing more robust results in the presence of electrode shifts.

Boschmann and Platzner (2014) utilized high density EMG signals and approached the EMG classification problem in a novel way. They translated the EMG recordings in images and used the luminance, contrast and structure of these images, in order to calculate a Structural Similarity Index (SSIM). SSIM quantifies the similarity between two images and is used to classify between 10 hand and wrist movements. The proposed classifier outperformed an LDA classifier in both shifted and unshifted data. The performance of the classifier was evaluated in the case of electrode displacement, but the same technique could generalize in the presence of other EMG disturbances, such as arm position.

### 2.3. Arm posture

A change in the posture of the patient's arm might result in different EMG recordings even when these are consistently measured from the same position on the subject's forearm muscles. Different hand postures might result in the same muscles to work differently with more or less effort, even if the hand performs the same grasp, either for limb stabilization or to counteract the effect of gravity (Scheme et al., 2010; Boschmann and Platzner, 2014; Gazzoni et al., 2014; Liu et al., 2014; Yang et al., 2015). Moreover different postures change the geometry of the muscles in shape or length, thus resulting to different myoelectrical excitation (Scheme et al., 2010; Liu et al., 2014).

More specifically, Gazzoni et al. (2014) investigated the effect of arm position in the sEMG activity distribution and they claim that along with electrode shift, these changes are due to gravity affecting in a different way on different body segments. They recorded data from subjects executing motions with their hand first in a neutral position and later in a prone position and found that the Center of Gravity (COG) of the sEMG activity areas is shifted by a 15*mm* inter-electrode distance (IED) for all considered motions.

In order to simulate the presence of variations of the EMG signal due to changes on arm posture many experiments involved performing the same grasp in different static pre-defined positions (Scheme et al., 2010; Chen et al., 2011; Fougner et al., 2011b; Khushaba et al., 2014; Betthauser et al., 2016), allowing the subject to perform a dynamic (Liu et al., 2012; Radmand et al., 2014; Yang et al., 2015) motion between pre-defined positions or free motion of the arm in the 3D space while executing a grasp (Castellini et al., 2009).

#### 2.3.1. Detection

As with the electrode displacement case, the result of changes in arm posture is quantified by recording the classification accuracy or classification error.

Liu et al. (2012) gathered EMG information from a static position of the arm (S) and during a dynamic motion of the arm (D), while executing a specific grasp. They trained two classifiers with data from the static condition and tested one with data from the static condition (S-S) and the other with data from the dynamic condition (S-D) and two classifiers trained in the dynamic condition and tested by data from the two conditions (D-S, D-D). The performance evaluation showed an increase in the average intra-set error (S-D and D-S cases) for all features.

Similar trends of increasing inter-position error of the same motion in different positions is observed throughout the literature (Scheme et al., 2010; Chen et al., 2011; Fougner et al., 2011b; Geng et al., 2012; Yang et al., 2015; Betthauser et al., 2016; Khushaba et al., 2016).

#### 2.3.2. Approaches for mitigation of drift due to arm posture

The most popular approach for dealing with variation due to differences in arm posture is to gather sufficient data that takes into account this variability, referred to as **data abundance**. In most cases this means recording data for each grasp in multiple arm postures and using a combination of those as training data for the classifier. This approach has proven successful in reducing the classification error in multiple studies (Scheme et al., 2010; Chen et al., 2011; Fougner et al., 2011b; Geng et al., 2012; Liu et al., 2012, 2014; Jiang et al., 2013; Khushaba et al., 2016).

Betthauser et al. (2016) showed that offline classification performance was degrading in asymmetric positions, which correspond to cases that real-world, testing data do not resemble the training conditions. However, they argue that training to as many positions as are needed for real-world use is not practical. To overcome this issue, they propose a sparse representation of the input data that successfully generalizes over different arm positions, achieving better performance than the LDA classifier.

Another approach in literature was to use sensors that provide information about the motion of the hand along with the EMG signals and incorporate the additional sensory information in the training data along EMG signals or using **cascade classifiers** to classify the arm position separately from the hand motion.

Scheme et al. (2010) trained a linear discriminant analysis (LDA) classifier using EMG recordings from 8 different positions and information from two 3D accelerometers to distinguish between eight motion classes. They trained the offline classifier with a combination of EMG and acceleration features and showed that the classifiers that included accelerometer data outperformed the EMG only classifiers in all positions. In a future study Fougner et al. (2011b) used accelerometers and EMG electrodes to train a classifier on five different limb positions and eight hand motion classes. When the accelerometers are added, two different schemes are investigated. The first has two separate states, one using accelerometer information to classify the limb position and one that decides on the grasp that was performed. The second is a one-stage classifier that is trained by a combination of features from accelerometers and EMG signals. Both schemes perform better than using only EMG information with the one-stage classifier being the best in terms of offline classification accuracy.

Geng et al. (2012) compared three different classifiers, one using information only from EMG from 5 different arm positions, one using EMG and tri-axial accelerometer mechanomyography (ACC-MMG) data from a single position and one two-stage classifier that uses firstly the ACC-MMG classifier to decide the arm position and in the second stage the EMG trained classifier to classify between 5 motions. In a real-time experiment they performed with able-bodied subjects, their results showed that the two-stage EMG-MMG classifier could significantly increase the average real-time completion rate, while achieving similar or a little better performance in the real-time motion response time, motion completion time, and dynamic efficiency.

These results show that adding extra sensory information seems beneficial to the performance of the classifier. However, Radmand et al. (2014) demonstrated that, unless the training data are collected across many positions, integrating acceleration information with EMG data can result in a worse performance than utilizing only EMG information. The result of adding data from newly seen positions is an increase of the grasp clusters' variance, hence a decrease in class separability, which affects the classifier's performance. Since training to all possible positions is not feasible and practical, they suggest collecting training data by moving the residual limb in a dynamic fashion through the region of interest.

Another aspect that is widely investigated is the robustness of different **feature sets** when the arm position changes. Liu et al. (2012) investigated the robustness of six different feature sets to arm posture changes. The features that were compared were time-domain features (TDS), 4th order autoregressive coefficients (AR4), 6th order autoregressive coefficients (AR6), the AR6 derived ceptrum coefficients and root mean square (RMS). They found a significant impact in the offline performance of the classifier for all the feature sets they tested (TDS, AR4, AR6, CA6, AR6+RMS, TDS+AR6+RMS) with the best performing classifier being the one trained with the combination of features (TDS+AR6+RMS).

Khushaba et al. (2012) compared the performance of a classifier utilizing a feature set based on a set of spectral moments with four other feature sets that are commonly used in literature. The proposed feature set performs best in the presence of arm posture changes between five different postures by 2.2% decrease of the offline average classification error.

In a subsequent study Khushaba et al. (2016) focused on the variability of the recorded EMG signal due to different orientations of the arm during the execution of a grasp. They performed a comparative study between different feature sets and showed that the time domain power spectral descriptors (TD-PSD) and discrete Fourier transform (DFT) features, which quantify the angle rather than the amplitude of the EMG signal, were the more robust in cases when the orientation of the arm changes. They also incorporated information from accelerometers and further improved the performance of the classifiers.

#### 2.3.3. Limitations

Not many experiments on the effect of arm posture involved amputee subjects. An important finding from research on amputees is that the changes in arm posture have less impact on signal deviation with amputee than with able-bodied subjects (Geng et al., 2012; Jiang et al., 2013). Anatomical changes in amputees' muscles due to shortened muscles and fixed limbs in prosthetic devices result in less variability, thus less dependence on changes in arm position. Nevertheless, the effect is big enough to cause a significant change in the classifier's behavior and it shouldn't be ignored in the design of a robust control scheme for prostheses.

Moreover, across literature the most popular method to ensure sufficient variability in the training data is to gather information from various limb positions, while executing a specific pre-grasp. This increases the duration of the training period which makes it more tiresome for the subjects and can lead to prosthesis abandonment. Some researchers have investigated the minimum amount of information that provides sufficient variability for a robust classification performance. Khushaba et al. (2012) have recorded EMG data from five different arm positions, but they argue that three positions are sufficient for acceptable performance. In a similar manner, Geng et al. (2012) observed that reducing the ACC-MMG channels from eight to two resulted in an increase of 0.3% on the average classification error.

However, simply gathering additional information from other sensors, like accelerometers does not necessarily lead to better performance if the testing data do not resemble the training data (Radmand et al., 2014). Hence, it is necessary to focus more on dynamic motions that correspond better to real-world usage of prosthesis devices when investigating the performance of classifiers in the precense of arm posture variability.

Finally most of the experiments evaluate the systems on offline performance and do not focus on real-time usage. As mentioned before, an increase in the performance of an offline classifier does not necessarily translate into a better online performance.

### 2.4. Intra-subject repeatability

Repeatability refers to the capability of using the same myoelectric control system over time. Here we are using intra-subject repeatability as an umbrella term that includes changes due to learning/adaptation of the user to the control of the prosthesis, but also for the general case of concept drift, which refers to a combination of different causes that could happen over the period of some time and affect the EMG signal.

#### 2.4.1. Learning/adaptation of the user

According to Fitts and Posner (1967) there are three stages of human motor learning;

1. The initial cognitive stage, which requires high mental load and movements are slow, inconsistent and inefficient,
2. The associative stage, which is characterized by lower conscious effort and higher performance, and
3. The autonomous stage, where the movements are accurate, consistent and efficient and they are performed unconsciously. Their characteristics are summarized in Table 2.

**Table 2:** Human motor learning stages and their motion and cognitive load characteristics.

**Learning stages**	**Motion characteristics**	**Cognitive load**
Cognitive	Slow, inconsistent, inefficient with large gains	High demand in cognitive load
Associative	Disjointed performance, more reliable & efficient, small gains	Requires less cognitive activity
Autonomous	Accurate, consistent, efficient, smooth	Unconscious - little or no cognitive load

After a subject is introduced to a new motor task the learning process starts and according to the time the subject spends on the task and the number of repetitions performed, the subject moves from one learning stage to the next. Since each stage is characterized by different motion execution, the EMG activity will also change following the cognitive learning. An alternative term used in literature to depict the changes, due to the fact that the user familiarizes to the new environmental or task requirements, is “user adaptation.”

##### 2.4.1.1. Detection

The main approach in literature to observe learning from the user involves experiments that span from a couple of days Ison et al. (2014) to 3 weeks Ison and Artemiadis (2015). Over this period data is recorded in regular basis and analyzed for trends that indicate the presence of learning from the user.

In a similar manner to the previous cases of EMG disturbance causes, the learning period of the user is manifested with higher classification error or lower classification accuracy in comparison to the initial performance of the classifier. The signals arriving to the classifier during the testing phase are different than the initial signals used to train the classifier, hence affecting the performance of the classifier.

A favored metric used in literature to detect disturbances is **entropy**. Entropy is a measure of confidence of a classification decision as a function of probability that a feature set belongs to each class. A decision with high entropy means low confidence and corresponds to the case where all classes have similar probabilities, whereas decisions with low entropy have high confidence as a result of clear differences in the classes probabilities.

Entropy has been used widely in the detection of user adaptation (Yokoi et al., 2004; Kato et al., 2006b; Ison et al., 2014). Yokoi et al. (2004) proposed a threshold rule that, when entropy measures stay below 0.14 the classification rate stays over 90%, in a classification application of 10 motion classes.

Besides entropy, other metrics used in literature include separability, repeatability and mean semi-principal axis indexes (He et al., 2013, 2015; Powell et al., 2014).

- **Separability** monitors the distance between different classes of one trial set. The Separability index (SI) is used to measure the diversity of different active motion classes.
- **Repeatability** measures how well subjects reproduced feature patterns. The repeatability index (RI) is used to measure the consistency of feature patterns of the same class among different days.
- **Mean semi-principal axis (MSA)** indicates the size of the class. Changes in individual cluster size correspond to changes in movement execution consistency.

Some researchers propose that the learning process stops after a specific amount of time that the user gets accustomed to the prosthesis, hence they focus on detecting the end of the learning period.

Zhang et al. (2008) performed an experiment over the period of 7 days and compared the performance of the classifier when using their proposed Optimized Wavelet Packet Energy Distribution (OWPED) method of extracting features vs. the AR coefficients features, proving that the former performed better. They also investigated the amount of data recording days that are needed in order to have a good performance of the classifier and observed that in the case of six motions classification 3 days was the optimum amount, since after these 3 days the recognition performance was only slightly changing.

He et al. (2015) performed a 12 hand and wrist motions classification experiment that lasted for 11 days. In order to compare the effects of learning over time they compared the offline *between-day classification error (BCE)* with the *within-day classification error (WCE)*. They observed that BCE was initially increasing in an exponential way but later it plateaued after 4 days for the able-bodied subjects and 9 days for the amputee subjects. The same trend was apparent in the RI (repeatability index). They argue that differences from day-to-day experiments could be the result of positioning the electrodes in slightly different positions with respect to the previous day, but this does not explain the overall decreasing and stabilizing trend that appears in the classification error. Based on this observation He et al. suggested that any adaptation algorithm should be applied after the user learning period, since it reaches a point where there is no significant difference.

Yokoi et al. (2004) and Kato et al. (2006a,b) used functional magnetic resonance imaging (f-MRI) in an attempt to detect changes in the human brain activity during learning. They repeated a classification myoelectric experiment for multiple days and compared the brain activity when EMG-to-motion classifier was performed in three cases; before any training took place, 3 h after the first training and after 1 month of training, which accounts as enough time for the user to have learned the task, since the number of motions that the real-time classifier could classify with a discrimination rate of over 80% had doubled from three to six after the training month. This trend was manifesting in the f-MRI by strongly activated primary motor area (M1) and primary somatosensory area (S1).

##### 2.4.1.2. User Learning Model

Existing models for the user adaptation in research approach the learning curve by fitting exponentially decaying models as functions of performance parameters, such as classification error and completion time.

He et al. (2015) propose an exponential function to fit the relationship between “between-day classification error” (BCE) and the evaluation sequence, as “user learning function”

$$\begin{array}{l} y=\alpha \exp^{\lambda x} \end{array}$$

where *y* and *x* represent the “between-day classification error” and the number of the evaluation sequence, respectively; α and λ are subject-dependent parameters of initial performance and learning rate respectively.

A similar model with the only difference being an added steady state value is proposed by Ison et al. (2014) and in Ison and Artemiadis (2015) the learning function in terms of completion time *c*_*t*_ for a trial *t* involves a sum of exponential decays:

$$\begin{array}{l} c_{t}=\alpha \exp^{\lambda t}+\beta \exp^{\kappa t} \end{array}$$

where the first additive component corresponds to an initial fast learning component and the second additive component to a slower long-term learning phase that follows the fast phase. Parameters β and κ correspond to the initial performance and learning rate of the slower learning phase respectively.

##### 2.4.1.3. User Knowledge Transfer

Although the majority of research in the implementation of myolectric human-robot interfaces focuses on the development of maximally intuitive systems, specifically regarding the user learning aspect, many scientists argue that intuitive tasks are not necessary and motions learned during a specific task can generalize and be used for the completion of a different task.

Work done by Mosier et al. (2005) involved subjects learning the mapping between the motion of a screen cursor and different finger motions. Subjects were able to learn the unintuitive mapping, but also reduced the motions performed in degrees of freedom that are not necessary to perform a motion as well as the variability of cursor and hand movements. A significant finding was that the subjects were able to generalize to new tasks that involved motions that were not included in the training session.

Pistohl et al. (2013) also proposed that the same myoelectric control scheme used to create the two dimensional muscle-cursor mapping can be transferred to real-life prosthetic applications, even when they are controlling a robotic hand with more degrees of freedom.

Ison et al. (Ison et al., 2014, 2016; Ison and Artemiadis, 2015) and Antuvan et al. (2014) argue that humans are able to explore the task space and learn the mapping between motions and tasks even when these are not straight forward and intuitively designed. They observe a natural emergence of a new muscle synergy space after multiple days of the user exploring the task space. Moreover they explored the possibility of generalization by learning one skill and performing equally well in a slightly different task. In Ison et al. (2014) and Antuvan et al. (2014) the subjects demonstrate knowledge transfer regarding the mapping function from one task to a new one, reducing the initial learning period of the new task. In Ison and Artemiadis (2015) the subjects are initially trained to control a virtual helicopter to reach four destinations or to teleoperate a robotic arm and after the learning period they were asked to perform the same task, but move the virtual helicopter to new position and teleoperate the robotic arm when the wrist was rotated respectively. The results showed that the subjects were able to generalize the control to the new tasks without requiring the initial learning curve. In a following study from Ison et al. (2016) the subjects learned to operate a virtual reality 7-DOF helicopter and after some days they used this knowledge to teleoperate a robot interface that uses the same controls as their training sessions to perform various grasping tasks. They modeled HD sEMG observations as mixture of activation signals and performed a muscle synergy-inspired decomposition to map myoelectric signals to control outputs. They observed that during learning the completion time of the task reduces, and the throughput and path efficiency increase. Notably these metrics are used by the Target Achievement Control (TAC) Test (Simon et al., 2011) and Fitt's law test (Scheme and Englehart, 2013) for the real-time evaluation of control performance. Ison et al. associated these adaptations with the dynamic formation of new muscle synergies, which allowed more efficient and precise control for the users over time.

Their approach did not require retraining or recalibration of the system between the different sessions neither the need of targeted electrode placement. They also argue that since there is no user-specific procedure their approach can potentially generalize across subjects, but they have not performed such an experiment. Moreover, none of their experiments involved amputee subjects.

#### 2.4.2. Concept drift

From a machine learning perspective “concept drift of the datastream” is the term that describes the changes over time in statistical properties of the target variable that the model is trying to predict. This results in less accurate predictions as time passes. In the case of myoelectric-based pattern recognition applications, “concept drift” is the result of fatigue, electrode displacement, user adaptation and many other factors that cause changes in EMG signals. In that respect, concept drift as it is conceptualized is not a cause but a symptom of various causes of signal drift. Many researchers consider concept drift as an outcome of various causes and attempt to mitigate it without necessarily identifying the individual causes. In that respect we consider also “concept drift” here in our review of causes of EMG signal drift as it is important to understand how researchers develop techniques to mitigate this phenomenon without identifying the actual underlying cause.

In general, two forms of concept drift have been described in literature,

1. Gradual concept drift and
2. Sudden (also referred to as fast, abrupt, instantaneous or drastic) concept drift (Tsymbal, 2004).

More specifically, according to Kato et al. (2006a), the gradual change in EMG signal properties is more correlated to physiological causes, such as muscle fatigue or skin impedance due to skin perspiration, and the drastic changes to physical reasons, such as electrode shift during usage.

Research on concept drift focuses on the detection of the changes to the EMG input and suggests solutions to deal with the classification accuracy degradation which is the result of these changes.

##### 2.4.2.1. Detection of Concept Drift

Concept drift is not the result of one single cause, thus signal disturbance detection is done via performance metrics, like classification accuracy, classification error or entropy (Kato et al., 2006a; Sensinger et al., 2009; Jain et al., 2012).

Kaufmann et al. (2010) monitored the offline classification accuracy over the period of 21 days and observed a gradual decrease over time. They associated this decrease with the combination of electrode movement and behavioral factors from the user corresponding to user adapting to the device.

Amsuss et al. (2013) have performed a 5 day repeatability experiment. Five subjects executed eight different hand motions and data was gathered over the period of the 5 days. Analysis on the data showed a decrease of the offline classification accuracy when the training and test data were from different days, most specifically there was a 4.1% decrease of classification accuracy per day. They identified three classes to hold a 76.5% of the total averaged misclassifications, hence requiring more attention in the training process.

Zhang and Huang (2015) suggested a sensor-fault-tolerant module (SFTM) and a self-recovery method to compensate for three signals disturbance causes: contact artifacts, loose contacts, and baseline noise. The SFTM calculates the Mahalanobis distance from the recorded data and each class model and if the new dataset has a large deviation from all the models it is characterized as disturbance. If the signal is not perceived as a result of disturbance it is added in the feature set and the classifier is retrained in real-time, in order to incorporate the new data. Their system was tested on able-bodied and amputee subjects and proved to sustain a high real-time classification accuracy in the presence of the aforementioned disturbances.

##### 2.4.2.2. Modeling of Concept Drift

As with the detection case, since concept drift is a combination of multiple reasons that result in EMG signal changes, there is no unified way of modeling the cause. In most cases data are gathered from the electrodes over a long period of hours or even days, in order to provide sufficient variability. Chen et al. (2013) gathered data from two separate trials in the same day for each subject, with an interval of 6–7 h. Phinyomark et al. (2012b) recorded data for 4 days and in a latter study Phinyomark et al. (2013a) 21 days, Liu et al. (2016a) for 10 days and Kaufmann et al. (2010) for 21 days. Models about the myoelectric activity are proposed, which are determined by the classifier used in each case. For example in the case of the LDA classifier implemented in Liu et al. (2016a) the model is characterized by the mean μ_*c*_ for each class *c* and the pooled covariance matrix ∑, which are the parameters that characterize the LDA classifier itself.

One attempt to model the disturbances in EMG recorded from leg muscles comes from the work by Huang et al. (2010). They used information about signal saturation manifested among other cases when EMG electrodes lose skin contact and simulated the drift and saturation of EMG signals by the following equation:

$$\begin{array}{l} y\left(i\right)=\left\{ \begin{array}{c} \alpha *PP\left(y\right),ify\left(i\right)\geq \alpha *PP\left(y\right)\;\\ y\left(i\right),ify\left(i\right)<\alpha *PP\left(y\right),\; \end{array}\right. \end{array}$$

where *y*(*i*) is the EMG signal recorded from one electrode; *PP*(*y*) denotes the peak-to-peak magnitude of EMG signal *y*(*i*) recorded in the experiment; and α is the signal drift level. A bigger α value corresponds to larger signal disturbance.

##### 2.4.2.3. Approaches for Mitigation of Drift Due to Combination of Causes

*2.4.2.3.1. Data abundance*. Bitzer and van der Smagt (2006) utilized an SVM classifier for the inter-session classification of finger movements. In order to include different arm postures in their experiment they gathered information from two different arm positions; relaxed and pronation. They achieve an average accuracy of 92% in the offline evaluation of their system.

Artemiadis and Kyriakopoulos (2011) allowed the subjects to move their hands freely in 3D space in order to use recorded information to control an anthropomorphic hand. This myoelectric information was used to train a switching control scheme. In the testing phase the classifier chooses between a discrete amount of models that correspond to different EMG disturbance levels. The switching classifier was compared with three different decoding schemes including a linear filter, an SVM classifier and a stationary model and outperformed them all, while maintaining the real-time accuracy in a stable level.

Kaufmann et al. (2010) gathered data from subjects for 21 days and compared the performance of five different pattern-matching algorithms in the classification task. For each of the algorithms they used training data

1. from all trials,
2. from the last five trials, and
3. from the first five trials.

All classifiers performed best when data from all the trials were used and worse when the least recent training data were used. This is also indicative of the user adaptation effect over time.

*2.4.2.3.2. Prosthesis-guided training (PGT)*. Simon et al. (2012) suggest a prosthesis-guided training that the user initiates whenever he feels that the performance of the prosthesis degrades. The prosthesis provides the cues by moving through a sequence of preprogrammed motions and the user imitates the prosthesis. This approach compensates also for the EMG changes originating from the differences in arm posture, displacement of the electrodes over use and due to the user learning the task over time. Since the execution of the training is user initiated, this does not seem to introduce unexpected device delays, when an automated algorithm decides to retrain, thus reducing possible frustration to the user. Lock et al. (2011) also took feedback from amputees using their PGT control system. The feedback suggested that PGT was perceived as an intuitive and desired feature by prosthesis user.

*2.4.2.3.3. Adaptation*. Training with all the data from all the days counteracts the effects of EMG disturbances, but continuously adding information to train a classifier soon becomes a very computationally expensive problem. Researchers tried to deal with this problem by adapting in an online manner to changes of the classifier and only selectively adding or eliminating training data. Patricia et al. (2014) have performed a comparative study of four different adaptation methods and proved the significance of adaptation vs. simple non-adaptive classifiers in all four cases.

One adaptive method used in research is referred to as **online incremental adaptation** and involves updating the classifier whenever the data gets outdated based on one of the detection metrics; most commonly classification accuracy, classification error or entropy.

Fukuda et al. (2003) utilized the entropy measure to evaluate the reliability of the classification output and use the most reliable data as feature set to retrain the classifier and update the weight of the log-linearized Gaussian mixture network (LLGMN) they are using to discriminate EMG patterns. The oldest data are removed from the system, in order to keep it updated to the latest EMG data resulting in a more stable system in comparison to the non-adapted approach. This approach was tested on a real-time manipulator control and included amputee subjects.

In a similar manner, Kato et al. (2006a,b) proposed an online EMG-to-motion discrimination system, which attempts to adapt to user's characteristics by managing learning data in real-time. In order to sustain a stable performance of the classifier over time they implemented three different methods, namely automatic elimination (AE), automatic addition (AA), selective addition (SA). The system monitors the classifier's performance over time, by calculating the continuity of motion, and adapts to gradual change by automatically eliminating (AE) or adding (AA) relevant training data and retraining the system. The SA is initiated by the user and adds new learning data, in order to compensate for more drastic changes in the classifier's performance. The classifier's performance is evaluated based on the time duration that a recognized motion is monitored, and any motion that is monitored within a window smaller than 0.22*s*, which is the average reaction time for a human (Laming., 1969), is perceived as a failed classification.

Sensinger et al. (2009) followed a similar procedure and compared the performance of supervised and unsupervised classifiers discriminating between eleven motion classes in real-time. The algorithm adds newly seen data to the training set when entropy is small, indicating high classification confidence or removes data that are not relevant any more, in order to correct errors. Every time that the training dataset changes the classifier needs to be retrained. They observed that supervised update of the classifier performs better than unsupervised, which is expected since the unsupervised method is more prone to errors and noise.

Chen et al. (2013) proposed a self-enhancing classifier that automatically incorporates new testing data in the existing classifier by updating the classifier's parameters. They investigated a self-enhancing LDA (SELDA) and a self-enhancing QDA (SEQDA) classifiers. When a new pattern from the testing set belonging to class *k* is acquired the model parameters are updated; for the SELDA the model parameters are the mean vector and pooled covariance matrix, and for the SEQDA these are the mean vector and class covariance matrix. The suggested enhanced algorithms showed an average improvement of 1.54 and 2.21% in the offline classification accuracy in the cases of LDA and QDA respectively. Most importantly the self-enhancing classifiers show less variability, which translates in more robust performance. They also compared the performance of the QDA and SEQDA classifiers across 14 testing cycles during the day and the self-enhancing classifier outperformed original QDA by 3.15%. The classification accuracy QDA on the long-term EMG data (9–11 h experiment) decreases over time, whereas for SEQDA the performance does not decrease much, indicating that the adaptive classifier is more robust for long-term use.

Vidovic et al. (2016) performed a 3 day online experiment in which a classifier was trained on the first day and the following days a small amount of calibration data was recorded from the subjects. The system's parameters were updated accordingly based on the newly acquired data. They proved that adaptation is highly beneficial in both offline and online experiments and for amputees as well as able-bodied subjects.

Gijsberts et al. (2014b) proposed a non-linear incremental learning method in which occasional updates utilizing an amount of novel training data allow continual adaptation to the changes in the signals. They ran a four session real-time experiment over the course of 2 days and they were able to perform stable myoelectric control of a hand prosthesis using non-linear incremental learning.

In an effect to automate the process Jain et al. (2012) proposed an algorithm that relies on an unsupervised as well as the on-demand update of the training set, and has been designed to adapt to both the slow and fast changes that occur in myoelectric signals. Concept drift is detected using the entropy measure and, in the case of slow drift the proposed algorithm updates the classifier by retraining with the newly recorded data, in order to follow the changes over time. In the case of fast concept drift a label correction algorithm is performed, which corrects the labels to be used in the new training set, helping maintain a consistently accurate classifier all throughout their experiments.

Liu (2015) have developed an unsupervised online incremental learning control scheme, where the classification result is treated as label for the newly seen data that are subsequently used to retrain an SVM classifier. The proposed unsupervised adaptive scheme proved to enhance the performance of the classifier over time. The experiment was performed only for 2 days.

Zhai et al. (2017) proposed a self-calibrating classifier that is automatically updated over time without the need of active retraining of the user for long-time use of prostheses. The proposed classifier is based on a deep convolutional neural network (CNN) that is trained using a combination of initial training data and a corrected version of the prediction results from previous testing sessions. Their results show an increase in classification accuracy of 10.18% for intact subjects and 2.99% for amputee subjects with respect to the unrecalibrated classifier. Comparing the performance of the classifier with that of an SVM classifier the proposed CNN-based system consistently showed better and more stable performance over time.

**Domain adaptation** is a specific aspect of transfer learning and refers to learning a well performing model from a source data distribution and applying it on a different (but related) target data distribution. Very recently Liu et al. (2016a) have performed a comparative study between two classifiers; namely the polynomial classifier and the LDA classifier, and the same classifiers when domain adaptation methods were applied. Their goal was to reduce the calibration time in day-to-day use of a prosthetic hand. They gathered EMG data from intact and amputee subjects over the period of 10 days and on each day they used the models from all the other 9 days to train nine models that were used as well as the current day's data.

In general, if $\bar{M}$ is the model from the current data and $\hat{M_{k}}$ is the model from he *kth* day, then the new model is formed as

$$\begin{array}{l} M=\left(1-r\right)\bar{M}+r\overset{p}{\underset{k=1}{\sum }}W_{k}\hat{M_{k}} \end{array}$$

Parameter *p* refers to the days beside the current day and *r* is a trade-off parameter between the model trained from current day data and the pre-trained data. Their results indicate that the performance with domain adaptation outperforms the non-adaptive algorithms, by raising the offline classification accuracy in a range of 5.49 to 28.48%, both in cases of intact and amputee subjects.

They also proposed a Common Model Component Analysis (CMCA) framework that performs an optimized projection of the training and testing data and tries to minimize the dissimilarity between different models. They trained the classifier using data from six different days and performed a motion test that simulated the real-time performance of the classifier on the computer (Liu et al., 2016b).

Du et al. (2017) proposed an unsupervised adaptation approach for inter-session sEMG-based gesture recognition based on a deep CNN. The classifier is continuously adapting to new data and they argue that it can be used for inter-session and inter-subject application. The suggested adaptation scheme achieved an average offline accuracy of 82.3% and an improvement of 19.6% in the inter-session recognition accuracy. An investigation was performed to evaluate the amount of calibration data required for a stable performance of the classifier and they observed that as little as 5% of calibration data is enough, which allows a fast calibration procedure.

##### 2.4.2.4. Limitations

Many of the proposed solutions to mitigate EMG disturbances require recording of a big amount of data during the day or over the period of different days. Training on different aspects is time demanding and needs pre-training with a lot of information which is computationally expensive (Artemiadis and Kyriakopoulos, 2010; Huang et al., 2010; Kaufmann et al., 2010).

On the other hand, the proposed online adaptation algorithms (Kato et al., 2006a; Sensinger et al., 2009; Huang et al., 2010) require less initial information, but the good performance of the classifiers relies on regular retraining, in order to update the classifier on the new input data. This adds an occasional delay while the prosthesis is on use, which can be frustrating for the users.

The domain adaptation approach (Liu et al., 2016a) attempts to reduce both the classification time and the necessity for huge amount of training data, while sustaining a high classification accuracy, but it has only been evaluated by offline measures, which does not necessarily translate into a good online performance.

## 3. EMG variability between subjects

Inter-subject generalization refers to the ability to produce a prosthetic hand control system that adapts to a new user with minimum or no training. The EMG signal is non-linear and varies significantly from one individual to another. Even though the underlying anatomy is the same, differences in anthropometric variables, like body mass and forearm circumference, or variations in the execution of the motions, due to individual preferences result in different EMG signals generation (De Luca, 1997).

An experiment on the effect of these variations in classification accuracy is reported by Castellini et al. (2009), with cross-subject average classification accuracy reaching 51.69 and 54.04% for still arm and free arm movements respectively, whereas the intra-subject classification accuracy was higher than 95%. This consists an important difference in performance, indicating the need for further investigation, in order to create devices that are easier to train and adapt to a novel user.

### 3.1. Approaches for inter-subject use

The different approaches in research involve gathering information from multiple subjects (**data abundance**) and evaluating the differences between them by either utilizing new **features** that minimize the presence of differences and maximizing the similarities or utilizing a **domain adaptation** method to adapt the newly read data to the known model/models.

In order to test how a classifier behaves for different users most research uses the leave-one-out approach where information is gathered from many subjects and subsequently the classifier is trained with data from all but one subject and tested on this specific subject (Matsubara et al., 2011; Gibson et al., 2013; Ison and Artemiadis, 2013; Matsubara and Morimoto, 2013; Guo et al., 2015; Park et al., 2016; Stival et al., 2016).

Gibson et al. (2013) gathered data from seven users and for the evaluation of performance of the classifier for each user they used a decision tree that uses variable thresholds trained on the data gathered from all the subjects except the one they were investigating. They achieved an overall real-time accuracy of 79 ± 6.6%, with average specificity (i.e., the likelihood of not predicting a given motion if the user is not performing that motion) of 97.6% and average sensitivity (i.e., the likelihood of predicting a given motion when the user is actually performing that motion) of 66%.

Besides gathering more information, research has also focused on the investigation of the existence of features or **feature sets** that describe better the motion and are more robust to individual differences. Phinyomark et al. (2013b) investigated the feasibility of using anthropometric variables, i.e., dimensions of the different parts of the body and physical characteristics like body mass in pattern-recognition based myoelectric control, and evaluated the correlation between the anthropometric variables and five common EMG features used in classification experiments. They suggested incorporating this information about correlations in the calibration of the controller, by calculating a weighting factor for the classifier and a normalizing value of EMG features based on the user's characteristics, but they have not yet published any online work that tests the performance of their suggested system.

Ison and Artemiadis (2013) used the discrete wavelet transform (DWT) method to perform a novel multi-resolution myscle synergy (MRMS) feature extraction. They recorded data from ten subjects and created a database which was then used to test the classifier's performance. For the training of the classifier they used data from all the subjects except the ones corresponding to the current user. They evaluated their results by calculating the area under the ROC plot (AUC) and the results suggested a very accurate classifier achieving classification accuracy of 92.4 ± 8.9%. These results, though, were only evaluated off-line.

Guo et al. (2015) performed a comparative study between four-dimensional time domain features (TD), 6th order autoreressive coefficients (AR) and a concatenation of them (TDAR) and showed that the latter performs best in an application of nine wrist and hand classification. They argue that their system can be used directly without any calibration or training from a new user with the only requirement being that the new user has similar physiological properties with the group used for the training. When they used TDAR features, for the real-time control performance, the offline classification accuracy (86%), real-time accuracy (83%), motion selection time (0.25*s*) and completion time (1.42*s*) for recognition of seven patterns are at a promising level.

### 3.2. Adaptation in inter-subject differences

Orabona et al. (2009) used a model adaptation approach, where they constructed a database of EMG signals from 10 different people for a classification task of 3 grasps, and created pre-trained models for each user utilizing the data from all the other subjects. When a new user appears the most similar pre-trained model was selected and used. Performance was evaluated by offline classification rates and the models obtained by adaptation proved to perform better compared to those trained using the training data from only the current user. This approach demands the storage of a lot of pre-trained models and requires a large amount of data to have a successful adaptation.

In a subsequent study Tommasi et al. (2013) use the Ninapro database (Atzori et al., 2014) to construct the pre-trained models and compare seven different adaptive and non-adaptive systems. They conclude that adaptive models outperform simpler models that are based on just gathering information from multiple users both in classification and regression cases. They also show that the classifier benefits from an adaptive method that consists of a linear combination of known models with different weight per class. They argue that the larger the amount of stored models is, the better the performance of the adaptive algorithms, although the use of prior models is only beneficial when there is a way to properly choose the best prior knowledge model and weigh and combine it with the newly acquired EMG data.

Chattopadhyay et al. (2011) utilizes the isomap feature which preserves the geodesic distance information between the distributions of different subjects and projects both training and unlabeled data on the same space. An experiment is performed to classify between the four combinations of low or high intensity of activity and low or high fatigue presence. They performed a comparative study between their topology preserving domain adaptation method with eight other methods from literature or variations of them and their suggested system outperformed them all to address subject based variability.

Matsubara et al. (2011) proposed a bilinear model that decomposes the EMG signal into two linear factors, one that is user dependent and one motion dependent and use the latter factor as user-independent features. They use information from multiple users, but in contrast to Orabona et al. (2009) they train and hold in memory a single bilinear model. They compare the performance of the adaptive classifier with a simple classifier trained with the data from multiple users and show that the former outperforms the latter by an average of 21% accuracy. They tested the real-time performance of their framework by controlling five motions to a three-fingered robotic hand. In a subsequent study Matsubara and Morimoto (2013) the newly seen subject is asked to demonstrate a few specific motions that are used to calibrate the model to their characteristics. They showed that their proposed model performs better in all cases, but for only up to three motions. Some limitation of their approach are that, in order for the limited calibration to work, they depend a lot in the precise placement of the electrodes and this is not realistic in cases of amputees with differences in the remaining limb. Thus their system is parameter dependent, since the dimensions of the style and content variables were experimentally selected by trial-and-error.

Khushaba (2014) also focus on the stylistic differences between subjects and proposed a parameter-free Canonical Correlation Analysis (CCA) model which involves the projection of both user and model data into the same space that maximizes their correlation coefficient. To this goal they also utilize time-domain derivation of spectral moments as features for their classifier as they were suggested in Khushaba et al. (2012). The new subjects are asked to perform one repetition of each predefined class for calibration purposes. Their proposed system achieves an average inter-subject offline accuracy of >82%, but the SVM classifier that uses the concatenated data from all-but-the-tested subject outperforms their proposed system. In the case of amputees though, the proposed system outperforms the SVM. The issue they face as in Matsubara and Morimoto (2013) is the variation in electrode placement due to differences in the amputees' limbs, which makes the comparison in same terms difficult.

Stival et al. (2016) proposed an online Gaussian Mixture Model framework, in order to adapt a model constructed from the pooled data from multiple users to a new user. They were able to provide good results when tested on a new user and proved that by updating the existing model by adding information gathered from the new subject improves the performance of their system. They used their proposed framework to control two grasps of a virtual prosthetic hand and the kick motion of a humanoid robot in real-time. In both cases though, the recognized motions are very simple and in the latter the amount of subjects is very low.

Côté-Allard et al. (2018) proposed a transfer learning approach for inter-user sEMG-based gesture recognition application based on a deep CNN. Deep learning methods require a large quantity of data, in order to train successfully, which would take an unreasonable amount of time for a single person to generate. In order to deal with this issue they are combining data from multiple subjects and train a user-independent network. Moreover, they attempt to model the effect of signal drifts, like fatigue, electrode displacement and noise, by augmenting the original dataset with artificial data that are manipulated to simulate the effect of each disturbance. Their suggested network achieves 98.31% offline classification accuracy for 7 hand/wrist gestures over 17 able-bodied participants.

### 3.3. Limitations

Research focused only recently on multi-subject prostheses, hence there is a limited number of real-time experiments (Matsubara et al., 2011; Matsubara and Morimoto, 2013; Guo et al., 2015; Stival et al., 2016). Moreover, the majority of the experiments involves able-bodied subjects and not amputees. Matsubara et al. (2011) and Matsubara and Morimoto (2013) have suggested that electrode placement influences the user dependent variables in their bilinear model and suggest to place the electrodes on specific muscles. This is difficult in the cases of amputees with different levels of amputation, thus it is necessary to include more amputee subjects in future experiments.

## 4. Discussion

This paper focuses on the reasons that cause significant variability in the EMG signal excitation over time, thus resulting in the deterioration of myoelectric based classifier performance. Muscle fatigue, electrodes displacement, arm posture and user adaptation have been identified as the main reasons behind this variability. Moreover, we report the effects and variability in the inter-subject cases. Different methods have been introduced in literature in order to mitigate their effects in the performance of the classifier. These include

1. information abundance, which refers to the process of gathering extra data from as many possible different configurations, in order to ensure that variability is sufficiently represented in the training data,
2. cascade classifiers, that as a first step determine the level of disturbance and as a second step classify the grasp performed,
3. incorporation of new sensors besides EMG, such as accelerometers,
4. investigation for robust feature sets, and
5. adaptation methods, that are able to monitor changes occurring in the EMG signal and mitigate their results.

The majority of myoelectric pattern recognition applications rely on gathering EMG data from various levels of disturbance, in an attempt to sufficiently capture the variability of the EMG signal over time. This approach has been proven beneficial for the classifier's performance in many cases, but is more demanding in capturing, storing and processing the dataset in comparison to a classifier that is trained in a simpler dataset. Sometimes, like in the case of HD-EMG systems or the multi-modal sensory systems that incorporate accelerometers, additional hardware is required, which results to design and energy consumption changes. The real-time performance of the prosthetic device, along with its weight are very important factors when it comes to patient's satisfaction with the device and continuation of usage (Biddiss and Chau, 2007), hence it is important to only incorporate new hardware when its benefits outperform the difficulties.

Feature selection is an important research topic and different features seem to be more beneficial in detecting or mitigating the effect of the various EMG drift causes. The presence of fatigue is best described by features that represent the EMG spectrum, specifically monitoring shifts toward the lower frequencies and the increase in signal amplitude. For the case of electrode shifts time-domain features that represent spatial patterns are proved to be more beneficial. In the case of arm posture variations, features that quantify the angle rather than the amplitude of EMG are more robust in arm orientation changes.

One new path in research is the application of domain adaptation techniques, such as transfer learning, for the mitigation of the aforementioned signal drift causes. Domain adaptation is based on the assumption that data under the presence of EMG drifts would be different than the training data, but also they would originate from the same distribution. When this is true, information gathered before the signal drift can be utilized to reduce the amount of time and data that are needed to adapt to the shifted signal. The majority of research on adaptive techniques shows that it can be beneficial in the case of EMG concept drift. The issues that rise in the case of domain adaptation consist of the processes of selection of which information is more relevant and which should be forgotten by the algorithm as outdated.

Recent advancements in deep learning research have provided great results in machine learning applications, especially in the fields of computer vision and speech recognition. This motivated the investigation of the suitability of deep learning methods for pattern recognition applications that are utilizing electromyographic data (Atzori et al., 2016; Du et al., 2017; Zhai et al., 2017). One interesting characteristic of deep convolutional networks is that the network can act like a feature extractor if it is deep enough, thus when it is used in a myoelectric pattern recognition application it removes the need to specify suitable features for the application (LeCun et al., 2015). Moreover, due to the nature of the training in a neural network, the process of transfer learning is very straightforward (Yosinski et al., 2014; LeCun et al., 2015). This behavior of deep networks indicates the necessity of further research, in order to evaluate the performance of such networks on myoelectric pattern recognition applications, that are dependent on the non-stationary EMG signal.

One important issue in literature is the limited amount of experiments with amputee subjects. Investigating how the different algorithms perform on able-bodied subjects provides important information, but it is necessary to gather information from amputees as well. Individual differences might be more in amount and quality in the cases of amputee subjects that have different levels of amputation.

Finally, there is lack of real-time experiments that involve able-bodied and amputee subjects manipulating the devices. Improving the offline performance is not enough to be beneficial for real-time use in a similar manner (Lock et al., 2005; Hargrove et al., 2007). Delays in response or classification mistakes during the real-time use can be interpreted by the users as their own mistakes or malfunctioning of the device causing user frustration. Since the acceptance of a prosthesis depends on the satisfaction of the user, these functionality issues could determine whether the user is going to continue wearing the prosthesis or not.

## Author contributions

IK has performed this literature review under the supervision of SV and ME. All authors listed contributed to the final version of the manuscript and approved it for publication.

### Conflict of interest statement

The authors declare that the research was conducted in the absence of any commercial or financial relationships that could be construed as a potential conflict of interest.

## Footnotes

^1^http://www.touchbionics.com/

^2^http://bebionic.com/

^3^https://vincentsystems.de/en/

^4^https://www.coaptengineering.com/

## References

[B1] Adewuyi A. A. Hargrove L. J. Kuiken T. A. (2016). Evaluating emg feature and classifier selection for application to partial-hand prosthesis control. Front. Neurorobot. 10:15. 10.3389/fnbot.2016.00015 27807418 PMC5069722

[B2] Ahsan M. Ibrahimy M. Khalifa O. (2011). Hand motion detection from emg signals by using ann based classifier for human computer interaction, in Modeling, Simulation and Applied Optimization (ICMSAO), 2011 4th International Conference on (Kuala Lumpur), 1–6.

[B3] Alkan A. Günay M. (2012). Identification of emg signals using discriminant analysis and svm classifier. Exp. Syst. Appl. 39, 44–47. 10.1016/j.eswa.2011.06.043

[B4] Al-Mulla M. R. Sepulveda F. (2010). Novel feature modelling the prediction and detection of semg muscle fatigue towards an automated wearable system. Sensors 10, 4838–4854. 10.3390/s100504838 22399910 PMC3292150

[B5] Al-Mulla M. R. Sepulveda F. Colley M. Kattan A. (2009). Classification of localized muscle fatigue with genetic programming on semg during isometric contraction, in 2009 Annual International Conference of the IEEE Engineering in Medicine and Biology Society (Minneapolis, MN), 2633–2638. 10.1109/IEMBS.2009.5335368 19965229

[B6] Amsuss S. Paredes L. P. Rudigkeit N. Graimann B. Herrmann M. J. Farina D. (2013). Long term stability of surface emg pattern classification for prosthetic control, in 2013 35th Annual International Conference of the IEEE Engineering in Medicine and Biology Society (EMBC) (Osaka), 3622–3625. 10.1109/EMBC.2013.6610327 24110514

[B7] Antuvan C. W. Ison M. Artemiadis P. (2014). Embedded human control of robots using myoelectric interfaces. IEEE Trans. Neural Syst. Rehabil. Eng. 22, 820–827. 10.1109/TNSRE.2014.2302212 24760930

[B8] Arendt-Nielsen L. Mills K. (1985). The relationship between mean power frequency of the EMG spectrum and muscle fibre conduction velocity. Electroencephalogr. Clin. Neurophysiol. 60, 130–134. 10.1016/0013-4694(85)90019-7 2578364

[B9] Artemiadis P. K. Kyriakopoulos K. J. (2008). Assessment of muscle fatigue using a probabilistic framework for an emg-based robot control scenario, in BioInformatics and BioEngineering, 2008. BIBE 2008. 8th IEEE International Conference on (Athens), 1–6.

[B10] Artemiadis P. K. Kyriakopoulos K. J. (2010). An EMG-based robot control scheme robust to time-varying EMG signal features. IEEE Trans. Inform. Technol. Biomed. 14, 582–588. 10.1109/TITB.2010.2040832 20172839

[B11] Artemiadis P. K. Kyriakopoulos K. J. (2011). A switching regime model for the EMG-based control of a robot arm. IEEE Trans. Syst. Man Cybern. B (Cybernetics) 41, 53–63. 10.1109/TSMCB.2010.2045120 20403787

[B12] Atzori M. Cognolato M. Mller H. (2016). Deep learning with convolutional neural networks applied to electromyography data: a resource for the classification of movements for prosthetic hands. Front. Neurorobot. 10:9. 10.3389/fnbot.2016.00009 27656140 PMC5013051

[B13] Atzori M. Gijsberts A. Castellini C. Caputo B. Hager A.-G. M. Elsig S.. (2014). Electromyography data for non-invasive naturally-controlled robotic hand prostheses. Sci. Data 1:140053. 10.1038/sdata.2014.53 25977804 PMC4421935

[B14] Bartuzi P. Roman-Liu D. (2014). Assessment of muscle load and fatigue with the usage of frequency and time-frequency analysis of the emg signal. Acta Bioeng. Biomech. 16, 31–39. 10.5277/abb140204 25088376

[B15] Basmajian J. V. De Luca C. (1985). Muscles Alive. Baltimore, MD: Williams & Wilkins.

[B16] Betthauser J. L. Hunt C. L. Osborn L. E. Kaliki R. R. Thakor N. V. (2016). Limb-position robust classification of myoelectric signals for prosthesis control using sparse representations, in 2016 38th Annual International Conference of the IEEE Engineering in Medicine and Biology Society (EMBC) (Orlando, FL), 6373–6376. 10.1109/EMBC.2016.7592186 PMC10947745 28325032

[B17] Biddiss E. Chau T. (2007). Upper-limb prosthetics: critical factors in device abandonment. Am. J. Phys. Med. Rehabil. 86, 977–987. 10.1097/PHM.0b013e3181587f6c 18090439

[B18] Bitzer S. van der Smagt P. (2006). Learning emg control of a robotic hand: towards active prostheses, in Proceedings 2006 IEEE International Conference on Robotics and Automation, 2006. ICRA 2006 (Orlando, FL), 2819–2823.

[B19] Bonato P. Roy S. H. Knaflitz M. De Luca C. J. (2001). Time-frequency parameters of the surface myoelectric signal for assessing muscle fatigue during cyclic dynamic contractions. IEEE Trans. Biomed. Eng. 48, 745–753. 10.1109/10.930899 11442286

[B20] Boschmann A. Platzner M. (2012). Reducing classification accuracy degradation of pattern recognition based myoelectric control caused by electrode shift using a high density electrode array, in 2012 Annual International Conference of the IEEE Engineering in Medicine and Biology Society (San Diego, CA), 4324–4327. 10.1109/EMBC.2012.6346923 23366884

[B21] Boschmann A. Platzner M. (2014). Towards robust hd emg pattern recognition: reducing electrode displacement effect using structural similarity, in Engineering in Medicine and Biology Society (EMBC), 2014 36th Annual International Conference of the IEEE (IEEE) (Chicago, IL), 4547–4550. 10.1109/EMBC.2014.6944635 25571003

[B22] Côté-Allard U. Fall C. L. Drouin A. Campeau-Lecours A. Gosselin C. Glette K. (2018). Deep learning for electromyographic hand gesture signal classification by leveraging transfer learning. arXiv:1801.07756 [Preprint]. 10.1109/TNSRE.2019.2896269 30714928

[B23] Camata T. V. Dantas J. L. Abro T. Brunetto M. A. O. C. Moraes A. C. Altimari L. R. (2010). Fourier and wavelet spectral analysis of emg signals in supramaximal constant load dynamic exercise, in 2010 Annual International Conference of the IEEE Engineering in Medicine and Biology (Buenos Aires), 1364–1367. 10.1109/IEMBS.2010.5626743 21096332

[B24] Cao H. Dib I. E. H. Antoni J. Marque C. (2007). Analysis of muscular fatigue during cyclic dynamic movement, in 2007 29th Annual International Conference of the IEEE Engineering in Medicine and Biology Society (Lyon), 1880–1883. 10.1109/IEMBS.2007.4352682 18002348

[B25] Castellini C. Fiorilla A. E. Sandini G. (2009). Multi-subject/daily-life activity emg-based control of mechanical hands. J. NeuroEng. Rehabil. 6, 1–11. 10.1186/1743-0003-6-41 19919710 PMC2784470

[B26] Castellini C. van der Smagt P. (2009). Surface emg in advanced hand prosthetics. Biol. Cybern. 100, 35–47. 10.1007/s00422-008-0278-1 19015872

[B27] Chan A. Englehart K. (2005). Continuous myoelectric control for powered prostheses using hidden markov models. IEEE Trans. Biomed. Eng. 52, 121–124. 10.1109/TBME.2004.836492 15651571

[B28] Chattopadhyay R. Krishnan N. C. Panchanathan S. (2011). Topology preserving domain adaptation for addressing subject based variability in semg signal, in AAAI Spring Symposium: Computational Physiology (Arlington, VA), 4–9.

[B29] Chen L. Geng Y. Li G. (2011). Effect of upper-limb positions on motion pattern recognition using electromyography, in Image and Signal Processing (CISP), 2011 4th International Congress on, Vol. 1 (Shanghai), 139–142.

[B30] Chen X. Zhang D. Zhu X. (2013). Application of a self-enhancing classification method to electromyography pattern recognition for multifunctional prosthesis control. J. NeuroEng. Rehabil. 10:44. 10.1186/1743-0003-10-44 23634939 PMC3689085

[B31] Christodoulou C. Pattichis C. (1999). Unsupervised pattern recognition for the classification of EMG signals. IEEE Trans. Biomed. Eng. 46, 169–178. 10.1109/10.740879 9932338

[B32] Cifrek M. Medved V. Tonković S. Ostojić S. (2009). Surface EMG based muscle fatigue evaluation in biomechanics. Clin. Biomech. 24, 327–340. 10.1016/j.clinbiomech.2009.01.010 19285766

[B33] Cobb S. Forbes A. (1923). Electromyographic studies of muscular fatigue in man. Amer. J. Physiol. 65, 234–251. 10.1152/ajplegacy.1923.65.2.234

[B34] Daley H. Englehart K. Hargrove L. Kuruganti U. (2012). High density electromyography data of normally limbed and transradial amputee subjects for multifunction prosthetic control. J. Electromyogr. Kinesiol. 22, 478–484. 10.1016/j.jelekin.2011.12.012 22269773

[B35] De Luca C. J. (1983). Myoelectrical manifestations of localized muscular fatigue in humans. Crit. Rev. Biomed. Eng. 11, 251–279. 6391814

[B36] De Luca C. J. (1997). The use of surface electromyography in biomechanics. J. Appl. Biomech. 13, 135–163. 10.1123/jab.13.2.135

[B37] De Luca C. J. Van Dyk E. J. (1975). Derivation of some parameters of myoelectric signals recorded during sustained constant force isometric contractions. Biophys. J. 15, 1167–1180. 10.1016/S0006-3495(75)85893-0 1203445 PMC1334801

[B38] Du Y. Jin W. Wei W. Hu Y. Geng W. (2017). Surface EMG-based inter-session gesture recognition enhanced by deep domain adaptation. Sensors 17:458. 10.3390/s17030458 28245586 PMC5375744

[B39] Enoka R. M. Duchateau J. (2007). Muscle fatigue: what, why and how it influences muscle function. J. Physiol. 586(Pt 1): 11–23. 10.1113/jphysiol.2007.139477 17702815 PMC2375565

[B40] Fang Y. Liu H. (2014). Robust semg electrodes configuration for pattern recognition based prosthesis control, in 2014 IEEE International Conference on Systems, Man, and Cybernetics (SMC) (San Diego, CA), 2210–2215.

[B41] Fitts P. M. Posner M. I. (1967). Human Performance. Belmont, CA: Brooks/Cole.

[B42] Fougner A. Scheme E. Chan A. D. Englehart K. Stavdahl Ø. (2011a). A multi-modal approach for hand motion classification using surface emg and accelerometers, in Engineering in Medicine and Biology Society, EMBC, 2011 Annual International Conference of the IEEE (IEEE), 4247–4250. 10.1109/IEMBS.2011.6091054 22255277

[B43] Fougner A. Scheme E. Chan A. D. C. Englehart K. Stavdahl O. (2011b). Resolving the limb position effect in myoelectric pattern recognition. IEEE Trans. Neural Syst. Rehabil. Eng. 19, 644–651. 10.1109/TNSRE.2011.2163529 21846608

[B44] Fukuda O. Tsuji T. Kaneko M. (1997). An EMG controlled robotic manipulator using neural networks, in Robot and Human Communication, 1997. RO-MAN '97. Proceedings., 6th IEEE International Workshop on (Sendai), 442–447.

[B45] Fukuda O. Tsuji T. Kaneko M. Otsuka A. (2003). A human-assisting manipulator teleoperated by EMG signals and arm motions. IEEE Trans. Robot. Automat. 19, 210–222. 10.1109/TRA.2003.808873

[B46] Gazzoni M. Celadon N. Mastrapasqua D. Paleari M. Margaria V. Ariano P. (2014). Quantifying forearm muscle activity during wrist and finger movements by means of multi-channel electromyography. PLoS ONE 9:e109943. 10.1371/journal.pone.0109943 25289669 PMC4188712

[B47] Geng Y. Zhou P. Li G. (2012). Toward attenuating the impact of arm positions on electromyography pattern-recognition based motion classification in transradial amputees. J. NeuroEng. Rehabil. 9, 1–11. 10.1186/1743-0003-9-74 23036049 PMC3551659

[B48] Gibson A. E. Ison M. R. Artemiadis P. (2013). User-independent hand motion classification with electromyography, in ASME 2013 Dynamic Systems and Control Conference (Stanford, CA: American Society of Mechanical Engineers).

[B49] Gijsberts A. Atzori M. Castellini C. Mller H. Caputo B. (2014a). Movement error rate for evaluation of machine learning methods for semg-based hand movement classification. IEEE Trans. Neural Syst. Rehabil. Eng. 22, 735–744. 10.1109/TNSRE.2014.2303394 24760932

[B50] Gijsberts A. Bohra R. Sierra Gonzlez D. Werner A. Nowak M. Caputo B.. (2014b). Stable myoelectric control of a hand prosthesis using non-linear incremental learning. Front. Neurorobot. 8:8. 10.3389/fnbot.2014.00008 24616697 PMC3935121

[B51] Guo W. Sheng X. Liu J. Hua L. Zhang D. Zhu X. (2015). Towards zero training for myoelectric control based on a wearable wireless semg armband, in 2015 IEEE International Conference on Advanced Intelligent Mechatronics (AIM) (Busan), 196–201.

[B52] Hägg G. (1981). Electromyographic fatigue analysis based on the number of zero crossings. Pflügers Archiv 391, 78–80. 10.1007/BF00580699 7279604

[B53] Hahne J. M. Farina D. Jiang N. Liebetanz D. (2016). A novel percutaneous electrode implant for improving robustness in advanced myoelectric control. Front. Neurosci. 10:114. 10.3389/fnins.2016.00114 27065783 PMC4814550

[B54] Hahne J. M. Graimann B. Muller K. (2012). Spatial filtering for robust myoelectric control. IEEE Trans. Biomed. Eng. 59, 1436–1443. 10.1109/TBME.2012.2188799 22374342

[B55] Hargrove L. Englehart K. Hudgins B. (2006). The effect of electrode displacements on pattern recognition based myoelectric control, in Engineering in Medicine and Biology Society, 2006. EMBS '06. 28th Annual International Conference of the IEEE (New York, NY), 2203–2206. 10.1109/IEMBS.2006.260681 17946096

[B56] Hargrove L. Englehart K. Hudgins B. (2008). A training strategy to reduce classification degradation due to electrode displacements in pattern recognition based myoelectric control. Biomed. Signal Process. Control 3, 175–180. 10.1016/j.bspc.2007.11.005

[B57] Hargrove L. Losier Y. Lock B. Englehart K. Hudgins B. (2007). A real-time pattern recognition based myoelectric control usability study implemented in a virtual environment, in 2007 29th Annual International Conference of the IEEE Engineering in Medicine and Biology Society (Lyon), 4842–4845. 10.1109/IEMBS.2007.4353424 18003090

[B58] Hargrove L. Zhou P. Englehart K. Kuiken T. A. (2009). The effect of ecg interference on pattern-recognition-based myoelectric control for targeted muscle reinnervated patients. IEEE Trans. Biomed. Eng. 56, 2197–2201. 10.1109/TBME.2008.2010392 19692302

[B59] He J. Zhang D. Jiang N. Sheng X. Farina D. Zhu X. (2015). User adaptation in long-term, open-loop myoelectric training: implications for emg pattern recognition in prosthesis control. J. Neural Eng. 12:046005. 10.1088/1741-2560/12/4/046005 26028132

[B60] He J. Zhang D. Sheng X. Zhu X. (2013). Effects of long-term myoelectric signals on pattern recognition, in International Conference on Intelligent Robotics and Applications (Berlin, Heidelberg: Springer), 396–404.

[B61] Huang H. Zhang F. Sun Y. L. He H. (2010). Design of a robust emg sensing interface for pattern classification. J. Neural Eng. 7:056005. 10.1088/1741-2560/7/5/056005 20811091 PMC2956305

[B62] Hudgins B. Parker P. Scott R. N. (1993). A new strategy for multifunction myoelectric control. IEEE Trans. Biomed. Eng. 40, 82–94. 10.1109/10.204774 8468080

[B63] Inbar G. F. Paiss O. Allin J. Kranz H. (1986). Monitoring surface EMG spectral changes by the zero crossing rate. Med. Biol. Eng. Comput. 24, 10–18. 10.1007/BF02441600 3959603

[B64] Ison M. Antuvan C. W. Artemiadis P. (2014). Learning efficient control of robots using myoelectric interfaces, in 2014 IEEE International Conference on Robotics and Automation (ICRA) (Hong Kong), 2880–2885.

[B65] Ison M. Artemiadis P. (2015). Proportional myoelectric control of robots: muscle synergy development drives performance enhancement, retainment, and generalization. IEEE Trans. Robot. 31, 259–268. 10.1109/TRO.2015.2395731

[B66] Ison M. Vujaklija I. Whitsell B. Farina D. Artemiadis P. (2016). High-density electromyography and motor skill learning for robust long-term control of a 7-dof robot arm. IEEE Trans. Neural Syst. Rehabil. Eng. 24, 424–433. 10.1109/TNSRE.2015.2417775 25838524

[B67] Ison M. R. Artemiadis P. (2013). Beyond user-specificity for emg decoding using multiresolution muscle synergy analysis, in ASME 2013 Dynamic Systems and Control Conference (Stanford, CA: American Society of Mechanical Engineers).

[B68] Jain S. Singhal G. Smith R. J. Kaliki R. Thakor N. (2012). Improving long term myoelectric decoding, using an adaptive classifier with label correction, in 2012 4th IEEE RAS EMBS International Conference on Biomedical Robotics and Biomechatronics (BioRob) (Rome), 532–537. 10.1109/biorob.2012.6290901 PMC10951549 38510572

[B69] Jiang N. Muceli S. Graimann B. Farina D. (2013). Effect of arm position on the prediction of kinematics from emg in amputees. Med. Biol. Eng. Comput. 51, 143–151. 10.1007/s11517-012-0979-4 23090099 PMC3581765

[B70] Jiang N. Vujaklija I. Rehbaum H. Graimann B. Farina D. (2014). Is accurate mapping of EMG signals on kinematics needed for precise online myoelectric control? IEEE Trans. Neural Syst. Rehabil. Eng. 22, 549–558. 10.1109/TNSRE.2013.2287383 24235278

[B71] Kato R. Fujita T. Yokoi H. Arai T. (2006a). Adaptable emg prosthetic hand using on-line learning method -investigation of mutual adaptation between human and adaptable machine, in ROMAN 2006 - The 15th IEEE International Symposium on Robot and Human Interactive Communication (Hatfield), 599–604.

[B72] Kato R. Yokoi H. Arai T. (2006b). Real-time learning method for adaptable motion-discrimination using surface EMG signal, in 2006 IEEE/RSJ International Conference on Intelligent Robots and Systems (Beijing), 2127–2132.

[B73] Kaufmann P. Englehart K. Platzner M. (2010). Fluctuating EMG signals: investigating long-term effects of pattern matching algorithms, in Engineering in Medicine and Biology Society (EMBC), 2010 Annual International Conference of the IEEE (Buenos Aires: IEEE), 6357–6360. 10.1109/IEMBS.2010.5627288 21096692

[B74] Khushaba R. N. (2014). Correlation analysis of electromyogram signals for multiuser myoelectric interfaces. IEEE Trans. Neural Syst. Rehabil. Eng. 22, 745–755. 10.1109/TNSRE.2014.2304470 24760933

[B75] Khushaba R. N. Al-Timemy A. Kodagoda S. Nazarpour K. (2016). Combined influence of forearm orientation and muscular contraction on emg pattern recognition. Exp. Syst. Appl. 61, 154–161. 10.1016/j.eswa.2016.05.031

[B76] Khushaba R. N. Shi L. Kodagoda S. (2012). Time-dependent spectral features for limb position invariant myoelectric pattern recognition, in Communications and Information Technologies (ISCIT), 2012 International Symposium on (Dallas, TX: IEEE), 1015–1020.

[B77] Khushaba R. N. Takruri M. Miro J. V. Kodagoda S. (2014). Towards limb position invariant myoelectric pattern recognition using time-dependent spectral features. Neural Netw. 55, 42–58. 10.1016/j.neunet.2014.03.010 24721224

[B78] Kim J.-S. Jeong H. Son W. (2004). A new means of HCI: EMG-mouse, in Systems, Man and Cybernetics, 2004 IEEE International Conference on (The Hague), Vol. 1, 100–104.

[B79] Krasoulis A. Kyranou I. Erden M. S. Nazarpour K. Vijayakumar S. (2017). Improved prosthetic hand control with concurrent use of myoelectric and inertial measurements. J. NeuroEng. Rehabil. 14:71. 10.1186/s12984-017-0284-4 28697795 PMC5505040

[B80] Kuiken T. A. Li G. Lock B. A. Lipschutz R. D. Miller L. A. Stubblefield K. A.. (2009). Targeted muscle reinnervation for real-time myoelectric control of multifunction artificial arms. JAMA 301, 619–628. 10.1001/jama.2009.116 19211469 PMC3036162

[B81] Kumar D. K. Pah N. D. Bradley A. (2003). Wavelet analysis of surface electromyography. IEEE Trans. Neural Syst. Rehabil. Eng. 11, 400–406. 10.1109/TNSRE.2003.819901 14960116

[B82] Kwatny E. Thomas D. H. Kwatny H. G. (1970). An application of signal processing techniques to the study of myoelectric signals. IEEE Trans. Biomed. Eng. BME-17, 303–313. 10.1109/TBME.1970.4502758 5518826

[B83] Kyranou I. Krasoulis A. Erden M. S. Nazarpour K. Vijayakumar S. (2016). Real-time classification of multi-modal sensory data for prosthetic hand control, in 2016 6th IEEE International Conference on Biomedical Robotics and Biomechatronics (BioRob) (Singapore), 536–541.

[B84] Laming D. R. J. (1969). Information, theory of choice-reaction times. new york: Academic press, 1968. Behav. Sci. 14, 330–333. 10.1002/bs.3830140408

[B85] LeCun Y. Bengio Y. Hinton G. (2015). Deep learning. Nature 521, 436–444. 10.1038/nature14539 26017442

[B86] Lindstrom L. Magnusson R. Petersen I. (1970). Muscular fatigue and action potential conduction velocity changes studied with frequency analysis of emg signals. Electromyography 10, 341–356. 5521836

[B87] Liu J. (2015). Adaptive myoelectric pattern recognition toward improved multifunctional prosthesis control. Med. Eng. Phys. 37, 424–430. 10.1016/j.medengphy.2015.02.005 25749182

[B88] Liu J. Sheng X. Zhang D. He J. Zhu X. (2016a). Reduced daily recalibration of myoelectric prosthesis classifiers based on domain adaptation. IEEE J. Biomed. Health Inform. 20, 166–176. 10.1109/JBHI.2014.2380454 25532196

[B89] Liu J. Sheng X. Zhang D. Jiang N. Zhu X. (2016b). Towards zero retraining for myoelectric control based on common model component analysis. IEEE Trans. Neural Syst. Rehabil. Eng. 24, 444–454. 10.1109/TNSRE.2015.2420654 25879963

[B90] Liu J. Zhang D. He J. Zhu X. (2012). Effect of dynamic change of arm position on myoelectric pattern recognition, in 2012 IEEE International Conference on Robotics and Biomimetics (ROBIO) (Guangzhou), 1470–1475.

[B91] Liu J. Zhang D. Sheng X. Zhu X. (2014). Quantification and solutions of arm movements effect on SEMG pattern recognition. Biomed. Signal Process. Control 13, 189–197. 10.1016/j.bspc.2014.05.001

[B92] Lock B. Englehart K. Hudgins B. (2005). Real-time myoelectric control in a virtual environment to relate usability vs. accuracy, in Myoelectric Symposium (Fredericton, NB).

[B93] Lock B. A. Simon A. M. Stubblefield K. Hargrove L. J. (2011). Prosthesis-guided training for practical use of pattern recognition control of prostheses, in Myoelectric Symposium (Fredericton, NB).

[B94] López N. M. di Sciascio F. Soria C. M. Valentinuzzi M. E. (2009). Robust emg sensing system based on data fusion for myoelectric control of a robotic arm. BioMed. Eng. OnLine 8:5. 10.1186/1475-925X-8-5 19243627 PMC2657216

[B95] Lucas M.-F. Gaufriau A. Pascual S. Doncarli C. Farina D. (2008). Multi-channel surface emg classification using support vector machines and signal-based wavelet optimization. Biomed. Signal Process. Control 3, 169–174. 10.1016/j.bspc.2007.09.002

[B96] Luttmann A. Jäger M. Laurig W. (2000). Electromyographical indication of muscular fatigue in occupational field studies. Int. J. Indust. Ergon. 25, 645–660. 10.1016/S0169-8141(99)00053-0

[B97] MacIsaac D. Parker P. A. Scott R. N. (2001). The short-time fourier transform and muscle fatigue assessment in dynamic contractions. J. Electromyogr. Kinesiol. 11, 439–449. 10.1016/S1050-6411(01)00021-9 11738956

[B98] Mainardi E. Urbano E. Davalli A. (2008). Design of a new EMG sensor for upper limb prosthetic control and real time frequency analysis, in Myoelectric Symposium (Fredericton, NB).

[B99] Masuda T. Miyano H. Sadoyama T. (1982). The measurement of muscle fiber conduction velocity using a gradient threshold zero-crossing method. IEEE Trans. Biomed. Eng. 10, 673–678. 10.1109/TBME.1982.324859 7173932

[B100] Matsubara T. Hyon S. H. Morimoto J. (2011). Learning and adaptation of a stylistic myoelectric interface: EMG-based robotic control with individual user differences, in 2011 IEEE International Conference on Robotics and Biomimetics (Phuket), 390–395.

[B101] Matsubara T. Morimoto J. (2013). Bilinear modeling of EMG signals to extract user-independent features for multiuser myoelectric interface. IEEE Trans. Biomed. Eng. 60, 2205–2213. 10.1109/TBME.2013.2250502 23475334

[B102] Merletti R. Knaflitz M. De Luca C. (1990). Myoelectric manifestations of fatigue in voluntary and electrically elicited contractions. J. Appl. Physiol. 69, 1810–1820. 10.1152/jappl.1990.69.5.1810 2272975

[B103] Merletti R. Sabbahi M. A. De Luca C. J. (1984). Median frequency of the myoelectric signal. Eur. J. Appl. Physiol. Occupat. Physiol. 52, 258–265. 10.1007/BF01015206 6539676

[B104] Mosier K. M. Scheidt R. A. Acosta S. Mussa-Ivaldi F. A. (2005). Remapping hand movements in a novel geometrical environment. J. Neurophysiol. 94, 4362–4372. 10.1152/jn.00380.2005 16148276

[B105] Muceli S. Jiang N. Farina D. (2014). Extracting signals robust to electrode number and shift for online simultaneous and proportional myoelectric control by factorization algorithms. IEEE Trans. Neural Syst. Rehabil. Eng. 22, 623–633. 10.1109/TNSRE.2013.2282898 24132017

[B106] Naik G. R. Nguyen H. T. (2015). Nonnegative matrix factorization for the identification of emg finger movements: evaluation using matrix analysis. IEEE J. Biomed. Health Inform. 19, 478–485. 10.1109/JBHI.2014.2326660 25486650

[B107] Naik G. R. Selvan S. E. Gobbo M. Acharyya A. Nguyen H. T. (2016). Principle component analysis applied to surface electromyography: a comprehensive review. IEEE Access. 4, 4025–4037. 10.1109/ACCESS.2016.2593013

[B108] Navaneethakrishna M. Ramakrishnan S. (2014). Multiscale feature based analysis of surface emg signals under fatigue and non-fatigue conditions, in 2014 36th Annual International Conference of the IEEE Engineering in Medicine and Biology Society (Chicago, IL), 4627–4630. 10.1109/EMBC.2014.6944655 25571023

[B109] Nishikawa D. Yu W. Maruishi M. Watanabe I. Yokoi H. Mano Y. (2000). On-line learning based electromyogram to forearm motion classifier with motor skill evaluation. JSME Int. J. Ser. C 43, 906–915. 10.1299/jsmec.43.906

[B110] Orabona F. Castellini C. Caputo B. Fiorilla A. E. Sandini G. (2009). Model adaptation with least-squares svm for adaptive hand prosthetics, in Robotics and Automation, 2009. ICRA '09. IEEE International Conference on (Kobe), 2897–2903.

[B111] Ortiz-Catalan M. Brånemark R. Håkansson B. (2013). Biopatrec: a modular research platform for the control of artificial limbs based on pattern recognition algorithms. Source Code Biol. Med. 8:11. 10.1186/1751-0473-8-11 23597283 PMC3669028

[B112] Ortiz-Catalan M. Rouhani F. Brånemark R. Håkansson B. (2015). Offline accuracy: a potentially misleading metric in myoelectric pattern recognition for prosthetic control, in 2015 37th Annual International Conference of the IEEE Engineering in Medicine and Biology Society (EMBC) (Milan), 1140–1143. 10.1109/EMBC.2015.7318567 26736467

[B113] Paiss O. Inbar G. F. (1987). Autoregressive modeling of surface emg and its spectrum with application to fatigue. IEEE Trans. Biomed. Eng. BME-34, 761–770. 10.1109/tbme.1987.325918 3679259

[B114] Pan L. Zhang D. Jiang N. Sheng X. Zhu X. (2015). Improving robustness against electrode shift of high density EMG for myoelectric control through common spatial patterns. J. Neuroeng. Rehabil. 12:110. 10.1186/s12984-015-0102-9 26631105 PMC4668610

[B115] Park E. Meek S. G. (1993). Fatigue compensation of the electromyographic signal for prosthetic control and force estimation. IEEE Trans. Biomed. Eng. 40, 1019–1023. 10.1109/10.247800 8294126

[B116] Park K. H. Suk H. I. Lee S. W. (2016). Position-independent decoding of movement intention for proportional myoelectric interfaces. IEEE Trans. Neural Syst. Rehabil. Eng. 24, 928–939. 10.1109/TNSRE.2015.2481461 26415203

[B117] Patricia N. Tommasit T. Caputo B. (2014). Multi-source adaptive learning for fast control of prosthetics hand, in Pattern Recognition (ICPR), 2014 22nd International Conference on (Stockholm: IEEE), 2769–2774.

[B118] Phinyomark A. Phukpattaranont P. Limsakul C. (2012a). Feature reduction and selection for emg signal classification. Exp. Syst. Appl. 39, 7420–7431. 10.1016/j.eswa.2012.01.102

[B119] Phinyomark A. Phukpattaranont P. Limsakul C. (2012b). Investigating long-term effects of feature extraction methods for continuous emg pattern classification. Fluctuat. Noise Lett. 11:1250028 10.1142/S0219477512500289

[B120] Phinyomark A. Quaine F. Charbonnier S. Serviere C. Tarpin-Bernard F. Laurillau Y. (2013a). EMG feature evaluation for improving myoelectric pattern recognition robustness. Exp. Syst. Appl. 40, 4832–4840. 10.1016/j.eswa.2013.02.023

[B121] Phinyomark A. Quaine F. Charbonnier S. Serviere C. Tarpin-Bernard F. Laurillau Y. (2013b). A feasibility study on the use of anthropometric variables to make muscle-computer interface more practical. Eng. Appl. Artif. Intell. 26, 1681–1688. 10.1016/j.engappai.2013.01.004

[B122] Phinyomark A. Thongpanja S. Hu H. Phukpattaranont P. Limsakul C. (2012c). The usefulness of mean and median frequencies in electromyography analysis, in Computational Intelligence in Electromyography Analysis-A Perspective on Current Applications and Future Challenges. London: InTech.

[B123] Pistohl T. Cipriani C. Jackson A. Nazarpour K. (2013). Abstract and proportional myoelectric control for multi-fingered hand prostheses. Ann. Biomed. Eng. 41, 2687–2698. 10.1007/s10439-013-0876-5 23934195 PMC3825263

[B124] Powell M. A. Kaliki R. R. Thakor N. V. (2014). User training for pattern recognition-based myoelectric prostheses: improving phantom limb movement consistency and distinguishability. IEEE Trans. Neural Syst. Rehabil. Eng. 22, 522–532. 10.1109/TNSRE.2013.2279737 24122566 PMC10497233

[B125] Putnam W. Knapp R. (1993). Real-time computer control using pattern recognition of the electromyogram, in Engineering in Medicine and Biology Society, 1993. Proceedings of the 15th Annual International Conference of the IEEE (Hong Kong), 1236–1237.

[B126] Radhakrishnan S. M. Baker S. N. Jackson A. (2008). Learning a novel myoelectric-controlled interface task. J. Neurophysiol. 100, 2397–2408. 10.1152/jn.90614.2008 18667540 PMC2576223

[B127] Radmand A. Scheme E. Englehart K. (2014). On the suitability of integrating accelerometry data with electromyography signals for resolving the effect of changes in limb position during dynamic limb movement. J. Prosthet. Orthot. 26, 185–193. 10.1097/JPO.0000000000000041

[B128] Ravier P. Buttelli O. Jennane R. Couratier P. (2005). An EMG fractal indicator having different sensitivities to changes in force and muscle fatigue during voluntary static muscle contractions. J. Electromyogr. Kinesiol. 15, 210–221. 10.1016/j.jelekin.2004.08.008 15664150

[B129] Sadoyama T. Masuda T. Miyano H. (1983). Relationships between muscle fibre conduction velocity and frequency parameters of surface emg during sustained contraction. Eur. J. Appl. Physiol. Occupat. Physiol. 51, 247–256. 10.1007/BF00455188

[B130] Scheme E. Fougner A. Stavdahl O. Chan A. D. C. Englehart K. (2010). Examining the adverse effects of limb position on pattern recognition based myoelectric control, in 2010 Annual International Conference of the IEEE Engineering in Medicine and Biology (Buenos Aires), 6337–6340. 10.1109/IEMBS.2010.5627638 21097173

[B131] Scheme E. J. Englehart K. B. (2013). Validation of a selective ensemble-based classification scheme for myoelectric control using a three-dimensional fitts' law test. IEEE Trans. Neural Syst. Rehabil. Eng. 21, 616–623. 23193252 10.1109/TNSRE.2012.2226189

[B132] Scheme E. J. Englehart K. B. Hudgins B. S. (2011). Selective classification for improved robustness of myoelectric control under nonideal conditions. IEEE Trans. Biomed. Eng. 58, 1698–1705. 10.1109/TBME.2011.2113182 21317073

[B133] Sensinger J. W. Lock B. A. Kuiken T. A. (2009). Adaptive pattern recognition of myoelectric signals: exploration of conceptual framework and practical algorithms. IEEE Trans. Neural Syst. Rehabil. Eng. 17, 270–278. 10.1109/TNSRE.2009.2023282 19497834 PMC3025709

[B134] Shin S. Langari R. Tafreshi R. (2014). A performance comparison of EMG classification methods for hand and finger motion, in ASME 2014 Dynamic Systems and Control Conference (San Antonio, CA: American Society of Mechanical Engineers).

[B135] Simon A. M. Hargrove L. J. Lock B. A. Kuiken T. A. (2011). The target achievement control test: evaluating real-time myoelectric pattern recognition control of a multifunctional upper-limb prosthesis. J. Rehabil. Res. Dev. 48:619 10.1682/JRRD.2010.08.0149 21938650 PMC4232230

[B136] Simon A. M. Lock B. A. Stubblefield K. A. (2012). Patient training for functional use of pattern recognition–controlled prostheses. J. Prosth. Orthot. 24:56. 10.1097/JPO.0b013e3182515437 22563231 PMC3339840

[B137] Song J.-H. Jung J.-W. Bien Z. (2006). Robust emg pattern recognition to muscular fatigue effect for human-machine interaction, in Mexican International Conference on Artificial Intelligence (Apizaco: Springer), 1190–1199.

[B138] Stango A. Negro F. Farina D. (2015). Spatial correlation of high density emg signals provides features robust to electrode number and shift in pattern recognition for myocontrol. IEEE Trans. Neural Syst. Rehabil. Eng. 23, 189–198. 10.1109/TNSRE.2014.2366752 25389242

[B139] Stival F. Michieletto S. Pagello E. (2016). Online subject-independent modeling of SEMG signals for the motion of a single robot joint, in 2016 6th IEEE International Conference on Biomedical Robotics and Biomechatronics (BioRob) (Singapore), 1110–1116.

[B140] Stulen F. B. Luca C. J. D. (1981). Frequency parameters of the myoelectric signal as a measure of muscle conduction velocity. IEEE Trans. Biomed. Eng. BME-28, 515–523. 10.1109/TBME.1981.324738 7275132

[B141] Subasi A. Kiymik M. K. (2010). Muscle fatigue detection in emg using time–frequency methods, ica and neural networks. J. Med. Syst. 34, 777–785. 10.1007/s10916-009-9292-7 20703933

[B142] Thongpanja S. Phinyomark A. Phukpattaranont P. Limsakul C. (2013). Mean and median frequency of emg signal to determine muscle force based on time-dependent power spectrum. Elektronika ir Elektrotechnika 19, 51–56. 10.5755/j01.eee.19.3.3697

[B143] Tkach D. Huang H. Kuiken T. A. (2010). Study of stability of time-domain features for electromyographic pattern recognition. J. Neuroeng. Rehabil. 7:21. 10.1186/1743-0003-7-21 20492713 PMC2881049

[B144] Tommasi T. Orabona F. Castellini C. Caputo B. (2013). Improving control of dexterous hand prostheses using adaptive learning. IEEE Trans. Robot. 29, 207–219. 10.1109/TRO.2012.2226386

[B145] Tsymbal A. (2004). The Problem of Concept Drift: Definitions and Related Work. Computer Science Department, Trinity College Dublin (Dublin), 106.

[B146] Vidovic M. M. C. Hwang H. J. Amsss S. Hahne J. M. Farina D. Mller K. R. (2016). Improving the robustness of myoelectric pattern recognition for upper limb prostheses by covariate shift adaptation. IEEE Trans. Neural Syst. Rehabil. Eng. 24, 961–970. 10.1109/TNSRE.2015.2492619 26513794

[B147] Vujaklija I. Roche A. D. Hasenoehrl T. Sturma A. Amsuess S. Farina D.. (2017). Translating research on myoelectric control into clinics are the performance assessment methods adequate? Front. Neurorobot. 11:7. 10.3389/fnbot.2017.00007 28261085 PMC5306214

[B148] Wei W. Wong Y. Du Y. Hu Y. Kankanhalli M. Geng W. (2017). A multi-stream convolutional neural network for semg-based gesture recognition in muscle-computer interface. Patt. Recogn. Lett. 10.1016/j.patrec.2017.12.005. [Epub ahead of print].

[B149] Yang D. Yang W. Huang Q. Liu H. (2015). Classification of multiple finger motions during dynamic upper limb movements. IEEE J. Biomed. Health Inform. 21, 134–141. 26469791 10.1109/JBHI.2015.2490718

[B150] Yokoi H. Arieta A. H. Katoh R. Yu W. Watanabe I. Maruishi M. (2004). Mutual adaptation in a prosthetics application, in Embodied Artificial Intelligence, eds Iida F. Pfeifer R. Steels L. Kuniyoshi Y. (Berlin; Heidelberg: Springer), 146–159.

[B151] Yokus M. A. Jur J. S. (2016). Fabric-based wearable dry electrodes for body surface biopotential recording. IEEE Trans. Biomed. Eng. 63, 423–430. 10.1109/TBME.2015.2462312 26241969

[B152] Yosinski J. Clune J. Bengio Y. Lipson H. (2014). How transferable are features in deep neural networks? CoRR abs/1411.1792.

[B153] Young A. J. Hargrove L. J. Kuiken T. A. (2012). Improving myoelectric pattern recognition robustness to electrode shift by changing interelectrode distance and electrode configuration. IEEE Trans. Biomed. Eng. 59, 645–652. 10.1109/TBME.2011.2177662 22147289 PMC4234037

[B154] Young A. J. Smith L. H. Rouse E. J. Hargrove L. J. (2013). Classification of simultaneous movements using surface EMG pattern recognition. IEEE Trans. Biomed. Eng. 60, 1250–1258. 10.1109/TBME.2012.2232293 23247839 PMC4208826

[B155] Zhai X. Jelfs B. Chan R. H. M. Tin C. (2017). Self-recalibrating surface EMG pattern recognition for neuroprosthesis control based on convolutional neural network. Front. Neurosci. 11:379. 10.3389/fnins.2017.00379 28744189 PMC5504564

[B156] Zhang D. Xiong A. Zhao X. Han J. (2012). PCA and LDA for emg-based control of bionic mechanical hand, in Information and Automation (ICIA), 2012 International Conference on (Shenyang), 960–965.

[B157] Zhang Q. Liu R. Chen W. Xiong C. (2017). Simultaneous and continuous estimation of shoulder and elbow kinematics from surface emg signals. Front. Neurosci. 11:280. 10.3389/fnins.2017.00280 28611573 PMC5447720

[B158] Zhang X. Chen X. Zhao Z.-Y. Li Q. Yang J.-H. Lantz V. (2008). An adaptive feature extractor for gesture SEMG recognition, in International Conference on Medical Biometrics (Berlin: Springer), 83–90.

[B159] Zhang X. Huang H. (2015). A real-time, practical sensor fault-tolerant module for robust EMG pattern recognition. J. NeuroEng. Rehabil. 12:18. 10.1186/s12984-015-0011-y 25888946 PMC4342209

[B160] Zhou P. Lowery M. M. Englehart K. B. Huang H. Li G. Hargrove L.. (2007). Decoding a new neural–machine interface for control of artificial limbs. J. Neurophysiol. 98, 2974–2982. 10.1152/jn.00178.2007 17728391

